# Synthesis, Characterization, and Catalytic Behaviors in Isoprene Polymerization of Pyridine–Oxazoline-Ligated Cobalt Complexes

**DOI:** 10.3390/polym16050578

**Published:** 2024-02-21

**Authors:** Xiuge Hao, Jin-Kui Liu, Weize Zhuo, Jiajing Zheng, Xin-Qi Hao, Jun-Fang Gong, Hui Jiang, Mao-Ping Song

**Affiliations:** College of Chemistry, Zhengzhou University, Zhengzhou 450001, China; 202012152012695@gs.zzu.edu.cn (X.H.); liujinkui28@gs.zzu.edu.cn (J.-K.L.); 202023000526@stu.zzu.edu.cn (W.Z.); zjj3412904822@stu.zzu.edu.cn (J.Z.); xqhao@zzu.edu.cn (X.-Q.H.); mpsong@zzu.edu.cn (M.-P.S.)

**Keywords:** pyridine–oxazoline ligand, cobalt catalyst, isoprene polymerization, isoprene

## Abstract

A family of pyridine–oxazoline-ligated cobalt complexes L_2_CoCl_2_ **3a**–**h** were synthesized and characterized. Determined via single-crystal X-ray diffraction, complexes **3a** and **3d,** ligated by two ligands, displayed a distorted tetrahedral coordination of a cobalt center. The X-ray structure indicated the pyridine–oxazoline ligands acted as unusual mono-dentate ligands by coordinating only to N_oxazoline_. Upon activation with AlEt_2_Cl (diethylaluminum chloride), these cobalt complexes all exhibited high catalytic activity (up to 2.5 × 10^6^ g·mol_Co_^−1^·h^−1^), affording *cis*-1,4-*co*-3,4-polyisoprene with molecular weights of 4.4–176 kg mol^−1^ and a narrow Ð of 1.79–3.42, suggesting a single-site nature of the active sites. The structure of cobalt catalysts and reaction parameters, especially co-catalysts and the reaction temperature, all have significant influence on the polymerization activity but not on the microstructure of polyisoprene.

## 1. Introduction

Due to the high demand for high-performance synthetic rubbers and the limited supply of natural rubbers, the importance of developing high-quality elastomers through conjugated diene polymerization has become increasingly prominent [1,2,3]. Isoprene is an attractive and versatile monomer, which could be stereospecifically incorporated in *tans*-1,4-, *cis*-1,4-, 1,2-, and 3,4- incorporation fashions in the polymerization process, the quantity and nature of which play a crucial role in its mechanical properties and application orientations [4,5]. Given its outstanding elastic properties, highly *cis*-1,4-polyisoprene exhibits marvelous flexibility and a low crystallinity, rendering it useful as an alternative to natural rubber [6], while 3,4-polyisoprene imparts enhanced wet skid resistance. In this context, the incorporation of 3,4-units into *cis*-1,4 enchainment, producing *cis*-1,4-*alt*-3,4- polyisoprene materials, is highly desirable to the rubber industry.

**Figure 1:**
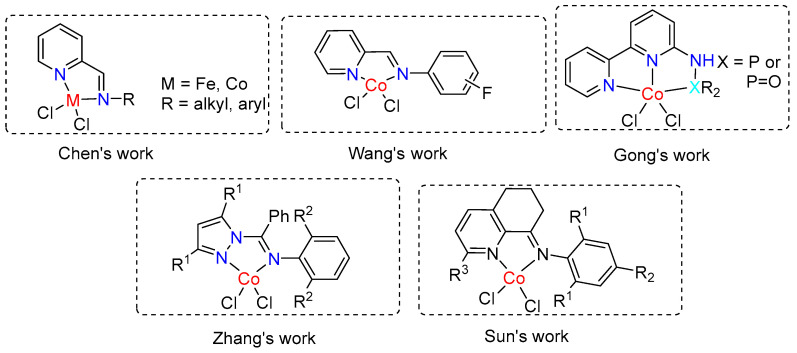
Cobalt complexes for isoprene polymerization.

Isoprene coordination–insertion polymerization catalyzed by a transition metal has witnessed great success in both industry practices and academia, since the introduction of the Ziegler–Natta catalyst. Thus, a great deal of novel high-activity and selective catalysts based on a wide range of late and early transition metals have been disclosed. The catalytic systems commonly used for isoprene polymerization are lithium, titanium, and lanthanide metal-based catalysts, which can effectively control the microstructure of isoprene polymers [7,8,9,10,11,12]. In particular, compared with lanthanide and early transition metals, cobalt complexes, as late transition metal catalysts [13,14,15], are extremely attractive, due to their low cost, ease of preparation, high moisture, and air stability, and have demonstrated high activity and versatile selectivity for conjugated diene polymerization, which therefore has attracted much attention for the large-scale polymer synthesis of conjugated dienes [16,17,18,19]. Therefore, more and more well-defined-structure cobalt complexes containing nitrogen ligands are being investigated in conjugated diene polymerization (Figure 1) [20,21,22,23,24,25,26,27,28,29,30]. In 2016, Chen’s group reported iminopyridine-ligated iron/cobalt complexes with different substituents in catalyzed isoprene polymerization to obtain polymers with different microstructures and molecular weights upon activation with methylaluminoxane (MAO) or ethylaluminum dichloride (AlEtCl_2_) [31]. In 2018, Gong’s group demonstrated that *cis*-1,4-*alt*-3,4 polyisoprene can be achieved with aminophosphine(ory)-fused bipyridine-based cobalt complexes in combination with MMAO [3]. Subsequently, Wang and coworkers investigated the effect of fluorine substituents on isoprene polymerization catalyzed by iminopyridine-supported cobalt catalysts activated by AlEt_2_Cl or MAO/[Ph_3_C][B(C_6_F_5_)_4_] [32,33,34]. The binary system exhibited high catalytic activity and *cis*-1,4 selectivity, and also obtained high-molecular-weight polyisoprene. However, the opposite trend was observed, i.e., the cobalt complexes displayed high *cis*-1,4 selectivity but gave a low-molecular-weight polymer, when replacing the binary system with a ternary system. Following these pioneering works of research, lots of cobalt complexes were developed to systemically investigate the effects of ligand skeletons on isoprene polymerization. In 2019, Zhang’s group showed that the cobalt complexes bearing pyrazolyl–imine displayed high activity in isoprene polymerization and afforded *cis*-1,4/3,4 polyisoprene under the activation of ethylaluminum sesquichloride (EASC) [35]. Very recently, Sun and coworkers reported that using rigid iminopyridine-ligated cobalt complexes for catalyzed isoprene polymerization can obtain a polymer with a high molecular weight and high *cis*-1,4 selectivity in the presence of AlEt_2_Cl [36].

**Scheme 1:**
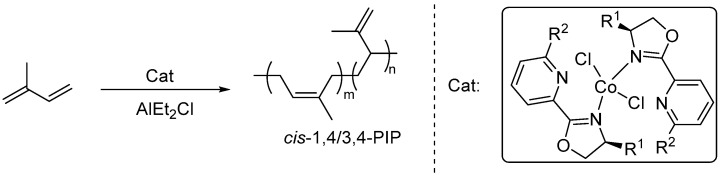
Pyridine–oxazoline-ligated cobalt complexes in catalyzed isoprene polymerization.

To the best of our knowledge, ligands coordinated to a metal have a very significant effect on the catalytic activity and microstructure of polymers. To further investigate the effect of the ligand structure, we designed and synthesized a series of cobalt complexes with special structures to prepare high-performance polyisoprene rubber. It is well known that chiral pyridine–oxazoline ligands are a relatively common and important class of ligands that have been widely used in asymmetric catalytic reactions [37,38,39]. However, reports of metal catalysts based on this ligand for olefin polymerization are relatively rare [40,41]. Following our continued research interests in this field [42,43], cobalt complexes supported by pyridine–oxazoline ligands with different chiral groups were synthesized to investigate their effect on the polymerization of isoprene (Scheme 1). Furthermore, the effects of the reaction parameters and ligand properties were studied in detail.

## 2. Experimental Section

### 2.1. General Conditions

All procedures and manipulations were carried out in an argon atmosphere by using a standard Schlenk line or a glovebox filling with argon unless otherwise mentioned. All solvents and monomers were heated to reflux temperature and distilled over sodium/benzophenone (toluene, tetrahydrofuran, and ether) or calcium hydride (CH_2_Cl_2_ and isoprene) in the argon atmosphere, then stored in the bottle with activated molecular sieves. Other reagents can be used directly without further purification.

^1^H, ^13^C{^1^H}, and ^19^F{^1^H} NMR spectrometry was performed on a Bruker 600 MHz instrument (Switzerland) with CDCl_3_ as the solvent and TMS as an internal standard at ambient temperature. Chemical shift multiplicities are represented as follows: s = singlet, d = doublet, t = triplet, q = quartet, m = multiplet, dd = doublet of doublets, td = triplet of doublets. The elemental analyses were recorded on a Flash Smart analyzer. HRMS for ligands was determined on a Waters Xevo G2-XS Q-Tof Micro LC/MS System ESI spectrometer operating in electrospray ionization mass spectrometry (ESI-MS) mode, and for cobalt complexes, on the Orbitrap Exploris 480 operating in matrix-assisted laser desorption/ionization time of flight mass spectrometry (MALDI-TOF-MS) mode. FT-IR spectra were collected with the PerkinElmer C100444. Optical rotations were recorded on a PerkinElmer 341 polarimeter. The X-ray diffraction analysis was conducted on a diffractometer with graphite-monochromated Mo and Cu K_α_ radiation. *M_n_* and Ð were determined via gel permeation chromatography (GPC) using a PL-220 equipped with two Agilent PLgel Olexis columns at 35 °C using THF as a solvent, with polystyrene as the standard and a flow rate of 1.0 mL/min.

### 2.2. General Procedure for the Synthesis of Ligands

**Synthesis of 6-Aryl-2-Picolinaldehyde 1:** The synthesis process was carried out according to the reported literature and the data obtained were consistent with those already reported [44]. Using commercial 6-bromo-2-picolinaldehyde (10 mmol, 1.0 eq) as the starting material, a coupling reaction with arylboronic acid (11 mmol, 1.1 eq) catalyzed by Pd(PPh_3_)_4_ (0.1 mmol, 1%) was carried out in toluene at reflux temperature. After 10 h, CH_2_Cl_2_ extracted the mixture and the obtained organic layer was dried over Na_2_SO_4_ and concentrated. The organic residue was purified with column chromatography on silica gel with petroleum ether/dichloromethane (1/1) as the eluent to obtain 6-aryl-2-picolinaldehyde **1**.

**6-phenylpicolinaldehyde (1a):** White solid (1.69 g, 92%). ^1^H NMR (600 MHz, CDCl_3_): *δ* 10.17 (s, 1H, C*H*O), 8.11–8.06 (m, 2H, Ar*H*), 7.98–7.87 (m, 3H, Ar*H*), 7.54–7.44 (m, 3H, Ar*H*) ppm. ^13^C{^1^H} NMR (151 MHz, CDCl_3_): *δ* 193.9, 157.9, 152.6, 138.1, 137.8, 129.7, 128.9, 127.0, 124.4, 119.8 ppm.

**6-(naphthalen-1-yl)picolinaldehyde (1b):** Yellow solid (1.75 g, 75%). ^1^H NMR (600 MHz, CDCl_3_): *δ* 10.18 (s, 1H, C*H*O), 8.07–7.91 (m, 5H, Ar*H*), 7.79 (dd, *J* = 7.4, 1.4 Hz, 1H, Ar*H*), 7.64 (dd, *J* = 7.0, 1.3 Hz, 1H, Ar*H*), 7.61–7.55 (m, 1H, Ar*H*), 7.55–7.46 (m, 2H, Ar*H*) ppm. ^13^C{^1^H} NMR (151 MHz, CDCl_3_): *δ* 193.8, 160.0, 152.7, 137.5, 137.3, 134.0, 131.0, 129.6, 129.3, 128.6, 127.8, 126.8, 126.2, 125.4, 125.2, 119.9 ppm.

**6-(4-(trifluoromethyl)phenyl)picolinaldehyde (1c):** White solid (2.24 g, 89%). ^1^H NMR (600 MHz, CDCl_3_): *δ* 10.17 (s, 1H, C*H*O), 8.22 (d, *J* = 8.2 Hz, 2H, Ar*H*), 8.03–7.93 (m, 3H, Ar*H*), 7.78 (d, *J* = 7.9 Hz, 2H, Ar*H*) ppm. ^13^C{^1^H} NMR (151 MHz, CDCl_3_): *δ* 193.7, 156.5, 153.1, 141.5, 138.3, 131.6 (q, ^2^*J*_C-F_ = 32.7 Hz), 127.5, 126.0 (q, ^3^*J*_C-F_ = 3.6 Hz), 124.8, 124.2 (q, ^1^*J*_C-F_ = 272.4 Hz), 120.7 ppm. ^19^F{^1^H} NMR (565 MHz, CDCl_3_): *δ* −62.68 ppm.

**6-(4-methoxyphenyl)picolinaldehyde (1d):** White solid (1.94 g, 91%). ^1^H NMR (600 MHz, CDCl_3_): *δ* 10.15 (s, 1H, C*H*O), 8.06 (d, *J* = 8.8 Hz, 2H, Ar*H*), 7.92–7.87 (m, 2H, Ar*H*), 7.84 (dd, *J* = 5.7, 3.0 Hz, 1H, Ar*H*), 7.04 (d, *J* = 8.9 Hz, 2H, Ar*H*), 3.89 (s, 3H, OC*H*_3_) ppm. ^13^C{^1^H} NMR (151 MHz, CDCl_3_): *δ* 194.2, 161.2, 157.7, 152.8, 137.8, 130.9, 128.5, 123.8, 119.2, 114.5, 55.5 ppm.

**General Procedure for the Synthesis of 2-Aryl-6-(oxazolinyl)pyridines 2:** According to the known literature, the procedures for the ligands **2** are as follows [45]: Chiral amino alcohol (11.0 mmol, 1.1 eq) was added to the tert-butanol solution of 6-Aryl-2-Picolinaldehyde (10 mmol, 1.0 eq) in an argon atmosphere and kept stirring at room temperature for 2 h. Then, K_2_CO_3_ (30 mmol, 3.0 eq) and I_2_ (20 mmol, 2.0 eq) were added subsequently to the above reaction system and stirred at 70 °C. After 16 h, saturated aqueous Na_2_S_2_O_3_ was added to quench the mixture until the color of the iodine disappeared. Then, CH_2_Cl_2_ extracted the mixture and the obtained organic layer was washed with brine, dried over Na_2_SO_4_, and concentrated. The organic residue was purified via column chromatography on silica gel with petroleum ether/ethyl acetate (10/1) as the eluent to obtain the ligands **2**. The analytical data of the compound are given as follows.

**(*S*)-4-benzyl-2-(6-phenylpyridin-2-yl)-4,5-dihydrooxazole (2a)**: White solid (1.92 g, 61%). ^1^H NMR (600 MHz, CDCl_3_): *δ* 8.07–8.00 (m, 3H, Ar*H*), 7.87–7.80 (m, 2H, Ar*H*), 7.49–7.40 (m, 3H, Ar*H*), 7.32 (t, *J* = 7.6 Hz, 2H, Ar*H*), 7.29–7.21 (m, 3H, Ar*H*), 4.72–4.64 (m, 1H, Ox*H*), 4.47 (dd, *J* = 9.5, 8.5 Hz, 1H, Ox*H*), 4.27 (dd, *J* = 8.5, 7.6 Hz, 1H, Ox*H*), 3.33 (dd, *J* = 13.8, 5.1 Hz, 1H, C*H*_2_Ph), 2.78 (dd, *J* = 13.8, 9.2 Hz, 1H, C*H*_2_Ph) ppm. ^13^C{^1^H} NMR (151 MHz, CDCl_3_): *δ* 163.6, 157.7, 146.9, 138.8, 138.0, 137.4, 129.4, 128.9, 128.7, 127.3, 126.7, 122.6, 122.5, 72.6, 68.3, 41.9 ppm.

**2-(6-phenylpyridin-2-yl)-4,5-dihydrooxazole (2a′):** White solid (1.54 g, 69%). ^1^H NMR (600 MHz, CDCl_3_): *δ* 8.07–8.03 (m, 2H, Ar*H*), 7.97 (dd, *J* = 6.1, 2.6 Hz, 1H, Ar*H*), 7.86–7.80 (m, 2H, Ar*H*), 7.49–7.38 (m, 3H, Ar*H*), 4.54 (t, *J* = 9.7 Hz, 2H, Ox*H*), 4.15 (t, *J* = 9.7 Hz, 2H, Ox*H*) ppm. ^13^C{^1^H} NMR (151 MHz, CDCl_3_): *δ* 164.2, 157.6, 146.8, 138.7, 137.4, 129.3, 128.8, 127.2, 122.33, 122.28, 68.3, 55.2 ppm. FT-IR (cm^−1^): 1636, 1565, 1449, 1367, 1267, 1244, 1110, 1062, 946, 823, 769, 739, 708, 691. HRMS (ESI-TOF) *m/z*: [M + Na]^+^ calcd for C_14_H_20_N_2_ONa^+^: 247.0842, found: 247.0839.

**(*S*)-4-benzyl-2-(6-(naphthalen-1-yl)pyridin-2-yl)-4,5-dihydrooxazole (2b):** Yellow solid (1.97 g, 54%). [α]^25^_D_ = −20.30 (*c* 0.109, CH_2_Cl_2_). ^1^H NMR (600 MHz, CDCl_3_): *δ* 8.16 (dd, *J* = 7.8, 1.1 Hz, 1H, Ar*H*), 8.01 (d, *J* = 8.4 Hz, 1H, Ar*H*), 7.94–7.88 (m, 2H, Ar*H*), 7.68 (dd, *J* = 7.8, 1.1 Hz, 1H, Ar*H*), 7.64 (dd, *J* = 7.0, 1.3 Hz, 1H, Ar*H*), 7.54 (dd, *J* = 8.2, 7.0 Hz, 1H, Ar*H*), 7.51–7.44 (m, 2H, Ar*H*), 7.32 (t, *J* = 7.5 Hz, 2H, Ar*H*), 7.30–7.22 (m, 2H, Ar*H*), 4.72–4.63 (m, 1H, Ox*H*), 4.45 (t, *J* = 9.0 Hz, 1H, Ox*H*), 4.5 (dd, *J* = 8.6, 7.6 Hz, 1H, Ox*H*), 3.32 (dd, *J* = 13.8, 5.1 Hz, 1H, C*H*_2_Ph), 2.79 (dd, *J* = 13.8, 9.1 Hz, 1H, C*H*_2_Ph) ppm. ^13^C{^1^H} NMR (151 MHz, CDCl_3_): *δ* 163.5, 159.3, 147.0, 138.0, 137.9, 136.9, 133.9, 131.4, 129.3, 129.1, 128.7, 128.4, 127.9, 127.1, 126.63, 126.59, 125.9, 125.5, 125.3, 122.5, 72.6, 68.2, 41.8 ppm. FT-IR (cm^−1^): 1639, 1567, 1453, 1250, 1109, 992, 961, 800, 776, 741, 700, 622. HRMS (ESI-TOF) *m*/*z*: [M + H]^+^ calcd for C_25_H_21_N_2_O^+^: 365.1648, found: 365.1653.

**(*S*)-4-benzyl-2-(6-(4-(trifluoromethyl)phenyl)pyridin-2-yl)-4,5-dihydrooxazole (2c):** White solid (2.37 g. 62%). [α]^25^_D_ = −46.26 (*c* 0.235, CH_2_Cl_2_). ^1^H NMR (600 MHz, CDCl_3_): *δ* 8.16 (d, *J* = 8.1 Hz, 2H, Ar*H*), 8.08 (dd, *J* = 7.5, 1.2 Hz, 1H, Ar*H*), 7.92–7.84 (m, 2H, Ar*H*), 7.73 (d, *J* = 8.2 Hz, 2H, Ar*H*), 7.32 (t, *J* = 7.5 Hz, 2H, Ar*H*), 7.29–7.22 (m, 3H, Ar*H*), 4.73–4.65 (m, 1H, Ox*H*), 4.48 (dd, *J* = 9.5, 8.5 Hz, 1H, Ox*H*), 4.28 (dd, *J* = 8.5, 7.6 Hz, 1H, Ox*H*), 3.32 (dd, *J* = 13.8, 5.1 Hz, 1H, C*H*_2_Ph), 2.79 (dd, *J* = 13.8, 9.1 Hz, 1H, C*H*_2_Ph) ppm. ^13^C{^1^H} NMR (151 MHz, CDCl_3_): *δ* 163.3, 156.2, 147.3, 142.1, 137.9, 137.7, 131.3 (q, ^2^*J*_C-F_ = 32.5 Hz), 129.4, 128.8, 127.7, 126.8, 125.8 (q, ^3^*J*_C-F_ = 3.8 Hz), 124.3 (q, ^1^*J*_C-F_ = 272.3 Hz), 123.4, 122.8, 77.4, 77.2, 77.0, 72.7, 68.3, 41.8 ppm. ^19^F{^1^H} NMR (565 MHz, CDCl_3_): *δ* −62.60 ppm. FT-IR (cm^−1^): 1641, 1564, 1458, 1368, 1327, 1162, 1105, 1070, 1051, 1014, 960, 862, 813, 753, 736, 703, 583. HRMS (ESI-TOF) *m*/*z*: [M + H]^+^ calcd for C_22_H_18_F_3_N_2_O^+^: 383.1366, found: 383.1375.

**(*S*)-4-benzyl-2-(6-(4-methoxyphenyl)pyridin-2-yl)-4,5-dihydrooxazole (2d):** White solid (2.24 g, 65%). [α]^25^_D_ = −32.50 (*c* 0.169, CH_2_Cl_2_). ^1^H NMR (600 MHz, CDCl_3_): *δ* 8.04–7.99 (m, 2H, Ar*H*), 7.96 (dd, *J* = 7.4, 1.3 Hz, 1H, Ar*H*), 7.84–7.75 (m, 2H, Ar*H*), 7.32 (t, *J* = 7.6 Hz, 2H, Ar*H*), 7.29–7.20 (m, 3H, Ar*H*), 7.02–6.96 (m, 2H, Ar*H*), 4.71–4.63 (m, 1H, Ox*H*), 4.46 (dd, *J* = 9.4, 8.5 Hz, 1H, Ox*H*), 4.27 (dd, *J* = 8.5, 7.5 Hz, 1H, Ox*H*), 3.86 (s, 3H, OC*H*_3_), 3.33 (dd, *J* = 13.8, 5.0 Hz, 1H, C*H*_2_Ph), 2.77 (dd, *J* = 13.8, 9.2 Hz, 1H, C*H*_2_Ph) ppm. ^13^C{^1^H} NMR (151 MHz, CDCl_3_): *δ* 163.7, 160.9, 157.4, 146.8, 138.1, 137.3, 131.5, 129.4, 128.8, 128.7, 126.7, 121.9, 121.8, 114.3, 72.6, 68.3, 55.5, 41.9 ppm. FT-IR (cm^−1^): 1644, 1606, 1563, 1512, 1450, 1368, 1296, 1243, 1114, 1025, 962, 809, 737, 705, 695, 656, 579. HRMS (ESI-TOF) *m*/*z*: [M + H]^+^ calcd for C_22_H_21_N_2_O_2_^+^: 345.1598, found: 345.1606.

**(*S*)-4-phenyl-2-(6-phenylpyridin-2-yl)-4,5-dihydrooxazole (2e):** White solid (1.77 g, 59%). ^1^H NMR (600 MHz, CDCl_3_): *δ* 8.15–8.10 (m, 1H, Ar*H*), 8.08–8.03 (m, 2H, Ar*H*), 7.87–7.81 (m, 2H, Ar*H*), 7.49–7.44 (m, 2H, Ar*H*), 7.42 (t, *J* = 7.3 Hz, 1H, Ar*H*), 7.39–7.33 (m, 4H, Ar*H*), 7.32–7.26 (m, 1H, Ar*H*), 5.47 (dd, *J* = 10.3, 8.5 Hz, 1H, Ox*H*), 4.92 (dd, *J* = 10.3, 8.5 Hz, 1H, Ox*H*), 4.41 (t, *J* = 8.5 Hz, 1H, Ox*H*) ppm. ^13^C{^1^H} NMR (151 MHz, CDCl_3_): *δ* 164.4, 157.7, 146.8, 142.1, 138.8, 137.5, 129.4, 128.9, 127.8, 127.3, 127.0, 122.8, 122.7, 75.5, 70.5 ppm.

**(*S*)-4-isopropyl-2-(6-phenylpyridin-2-yl)-4,5-dihydrooxazole (2f):** White solid (1.73 g, 65%). ^1^H NMR (600 MHz, CDCl_3_): *δ* 8.07–8.01 (m, 3H, Ar*H*), 7.85–7.79 (m, 2H, Ar*H*), 7.49–7.44 (m, 2H, Ar*H*), 7.44–7.39 (m, 1H, Ar*H*), 4.54 (dd, *J* = 9.7, 8.3 Hz, 1H, Ox*H*), 4.25 (t, *J* = 8.3 Hz, 1H, Ox*H*), 4.21–4.14 (m, 1H, Ox*H*), 1.96–1.87 (m, 1H, C*H*(CH_3_)_2_), 1.08 (d, *J* = 6.8 Hz, 3H, CH(C*H*_3_)_2_), 0.96 (d, *J* = 6.8 Hz, 3H, CH(C*H*_3_)_2_) ppm. ^13^C{^1^H} NMR (151 MHz, CDCl_3_): *δ* 162.9, 157.5, 146.2, 139.3, 137.2, 129.2, 128.7, 127.2, 122.5, 122.3, 72.9, 70.8, 32.9, 19.1, 18.2 ppm.

**(*S*)-4-benzyl-2-(6-bromopyridin-2-yl)-4,5-dihydrooxazole (2g):** White solid (1.93 g, 61%). ^1^H NMR (600 MHz, CDCl_3_): *δ* 8.03 (dd, *J* = 7.4, 1.1 Hz, 1H, Ar*H*), 7.67–7.58 (m, 2H, Ar*H*), 7.34–7.28 (m, 2H, Ar*H*), 7.26–7.21 (m, 3H, Ar*H*), 4.69–4.61 (m, 1H, Ox*H*), 4.45 (dd, *J* = 9.5, 8.6 Hz, 1H, Ox*H*), 4.24 (dd, *J* = 8.6, 7.7 Hz, 1H, Ox*H*), 3.28 (dd, *J* = 13.8, 5.2 Hz, 1H, C*H*_2_Ph), 2.75 (dd, *J* = 13.8, 9.0 Hz, 1H, C*H*_2_Ph) ppm. ^13^C{^1^H} NMR (151 MHz, CDCl_3_): *δ* 162.0, 147.7, 142.0, 138.8, 137.6, 130.4, 129.2, 128.6, 126.7, 122.9, 72.7, 68.2, 41.6 ppm.

**(*S*)-4-benzyl-2-(pyridin-2-yl)-4,5-dihydrooxazole (2h):** White solid (1.33 g, 56%). ^1^H NMR (600 MHz, CDCl_3_): *δ* 8.69 (d, *J* = 4.8 Hz, 1H, Ar*H*), 8.04 (d, *J* = 7.9 Hz, 1H, Ar*H*), 7.76 (td, *J* = 7.7, 1.8 Hz, 1H, Ar*H*), 7.44–7.35 (m, 1H, Ar*H*), 7.34–7.13 (m, 5H, Ar*H*), 4.68–4.60 (m, 1H, Ox*H*), 4.42 (dd, *J* = 9.4, 8.6 Hz, 1H, Ox*H*), 4.21 (t, *J* = 8.1 Hz, 1H, Ox*H*), 3.28 (dd, *J* = 13.8, 5.1 Hz, 1H, C*H*_2_Ph), 2.74 (dd, *J* = 13.8, 9.1 Hz, 1H, C*H*_2_Ph) ppm. ^13^C{^1^H} NMR (151 MHz, CDCl_3_): *δ* 162.8, 149.5, 146.5, 137.5, 136.4, 129.0, 128.3, 126.3, 125.3, 123.7, 72.2, 67.8, 41.4 ppm.

### 2.3. General Procedure for the Synthesis of Cobalt Complexes

All of the cobalt complexes were synthesized in a similar method. A classical synthesis process of the complex **3a** is described as follows: In a glovebox, the corresponding ligand **2a** (0.31 g, 1.0 mmol), anhydrous CoCl_2_ (65 mg, 0.5 mmol), and freshly distilled THF (10 mL) were added subsequently to a 25 mL Schlenk bottle. The mixture was stirred for 24 h at ambient temperature. The product was obtained by removing the solvents, filtered, washed with dry diethyl ether three times, and then dried under vacuum at 40 °C for 24 h.

**(*S*)-4-benzyl-2-(6-phenylpyridin-2-yl)-4,5-dihydrooxazole cobalt chloride (3a):** Blue solid (315 mg, 83%). [α]^30^_D_ = +69.62 (*c* 0.26, CH_2_Cl_2_). FT-IR (cm^−1^): 1652, 1601, 1579, 1445, 1425, 1375, 1249, 1173, 954, 822, 770, 748, 738, 723, 676, 624. Anal. Calcd for C_42_H_36_Cl_2_CoN_4_O_2_: C, 66.50; H, 4.78; N, 7.39. Found: C, 66.53; H, 4.54; N, 7.12. HRMS (MALDI-TOF) *m*/*z*: [M−Cl]^+^ calcd for C_42_H_36_ClCoN_4_O_2_^+^: 722.1853, found: 722.1847.

**2-(6-phenylpyridin-2-yl)-4,5-dihydrooxazole cobalt chloride (3a′):** Blue solid. (223 mg, 77%). FT-IR (cm^−1^): 1673, 1591, 1481, 1438, 1381, 1251, 1183, 927, 827, 768, 750, 719, 703. Anal. Calcd for C_28_H_24_Cl_2_CoN_4_O_2_: C, 58.15; H, 4.18; N, 9.69. Found C, 58.30; H, 4.02; N, 9.34. HRMS (MALDI-TOF) *m*/*z*: [M−Cl]^+^ calcd for C_28_H_24_ClCoN_4_O_2_^+^: 542.0914, found: 542.0911.

**(*S*)-4-benzyl-2-(6-(naphthalen-1-yl)pyridin-2-yl)-4,5-dihydrooxazole cobalt chloride (3b).** Blue solid (364 mg, 85%). [α]^30^_D_ = −25.65 (*c* 0.50, CH_2_Cl_2_). FT-IR (cm^−1^): 1646, 1587, 1469, 1446, 1432, 1380, 1251, 1181, 938, 804, 779, 741, 706, 676, 627. Anal. Calcd for C_50_H_40_Cl_2_CoN_4_O_2_: C, 69.93; H, 4.70; N, 6.52. Found: C, 70.18; H, 4.65; N, 6.40. HRMS (MALDI-TOF) *m*/*z*: [M−Cl]^+^ calcd for C_50_H_40_ClCoN_4_O_2_^+^: 822.2166, found: 822.2126.

**(*S*)-4-benzyl-2-(6-(4-(trifluoromethyl)phenyl)pyridin-2-yl)-4,5-dihydrooxazole cobalt chloride (3c).** Blue solid. (389 mg, 87%). [α]^30^_D_ = +64.41 (*c* 0.25, CH_2_Cl_2_). FT-IR (cm^−1^): 1653, 1586, 1441, 1323, 1248, 1167, 1119, 1078, 1058, 1016, 953, 858, 815, 751, 710, 684. Anal. Calcd for C_44_H_34_Cl_2_CoF_6_N_4_O_2_: C, 59.07; H, 3.83; N, 6.26. Found: C, 59.05; H, 3.78; N, 6.15.

**(*S*)-4-benzyl-2-(6-(4-methoxyphenyl)pyridin-2-yl)-4,5-dihydrooxazole cobalt chloride (3d).** Blue solid (332 mg, 81%). [α]^30^_D_ = +101.56 (*c* 0.26, CH_2_Cl_2_). FT-IR (cm^−1^): 1649, 1607, 1591, 1517, 1438, 1379, 1249, 1179, 1023, 934, 847, 815, 747, 707, 582. Anal. Calcd for C_44_H_40_Cl_2_CoN_4_O_4_: C, 64.55; H, 4.93; N, 6.84. Found: C, 64.50; H, 4.86; N, 6.79.

**(*S*)-4-phenyl-2-(6-phenylpyridin-2-yl)-4,5-dihydrooxazole cobalt chloride (3e).** Blue solid (267 mg, 73%). [α]^30^_D_ = +69.62 (*c* 0.26, CH_2_Cl_2_). FT-IR (cm^−1^): 1644, 1589, 1451, 1435, 1380, 1253, 1186, 929, 828, 764, 699. Anal. Calcd for C_40_H_32_Cl_2_CoN_4_O_2_: C, 65.76; H, 4.42; N, 7.67. Found: C, 65.31; H, 4.49; N, 7.52. HRMS (MALDI-TOF) *m*/*z*: [M−Cl]^+^ calcd for C_40_H_32_ClCoN_4_O_2_^+^: 694.1540, found: 694.1525.

**(*S*)-4-isopropyl-2-(6-phenylpyridin-2-yl)-4,5-dihydrooxazole cobalt chloride (3f).** Blue solid (249 mg, 75%). [α]^30^_D_ = +78.50 (*c* 0.25, CH_2_Cl_2_). FT-IR (cm^−1^): 1643, 1589, 1448, 1380, 1258, 1188, 1089, 1011, 928, 834, 768, 755, 734, 701. Anal. Calcd for C_34_H_36_Cl_2_CoN_4_O_2_: C, 61.64; H, 5.48; N,8.46. Found: C, 61.54; H, 5.42; N, 8.35. HRMS (MALDI-TOF) *m*/*z*: [M−Cl]^+^ calcd for C_34_H_36_ClCoN_4_O_2_^+^: 626.1853, found: 626.1852.

**(*S*)-4-benzyl-2-(6-bromopyridin-2-yl)-4,5-dihydrooxazole cobalt chloride (3g).** Blue solid (275 mg, 72%). [α]^30^_D_ = +43.86 (*c* 0.30, CH_2_Cl_2_). FT-IR (cm^−1^): 1632, 1572, 1454, 1420, 1381, 1256, 1180, 1002, 948, 934, 864, 815, 756, 748, 702, 659. Anal. Calcd for C_30_H_28_Br_2_Cl_2_CoN_4_O_2_: C, 47.15; H, 3.43; N, 7.33. Found: C, 47.32; H, 3.36; N, 7.25. HRMS (MALDI-TOF) *m*/*z*: [M−Cl]^+^ calcd for C_30_H_26_Br_2_ClCoN_4_O_2_^+^: 725.9438, found: 725.9429.

**(*S*)-4-benzyl-2-(pyridin-2-yl)-4,5-dihydrooxazole cobalt chloride (3h).** Blue solid (246 mg, 81%). [α]^30^_D_ = +42.11 (*c* 0.26, EtOH). FT-IR (cm^−1^): 1652, 1594, 1493, 1401, 1300, 1260, 1148, 1050, 1017, 955, 935, 798, 762, 748, 723, 704, 674, 639. Anal. Calcd for C_30_H_28_Cl_2_CoN_4_O_2_: C, 59.42; H, 4.65; N, 9.24. Found: C, 59.17; H, 4.71; N, 9.18. HRMS (MALDI-TOF) *m*/*z*: [M−Cl]^+^ calcd for C_30_H_28_ClCoN_4_O_2_^+^: 570.1227, found: 570.1213.

### 2.4. General Procedure for Isoprene Polymerization

The general procedure for isoprene polymerization is as follows: In a glovebox, the cobalt complexes (5 μmol) and toluene (5 mL) were added to an oven-dried 25 mL Schlenk bottle, stirred for 2 min, then AlEt_2_Cl was added and stirred for 2 min. Finally, isoprene (2 mL) was injected into the mixture with continuous stirring. After a certain period of time, acidic EtOH (5% HCl in EtOH) was added to quench the reaction. The resultant polymers were collected, washed with EtOH repeatedly, and dried in a vacuum oven at 50 °C to reach a constant weight. The conversion rate was obtained via the weight ratio of the resulting polyisoprene to the used isoprene.

## 3. Results and Discussion

### 3.1. Characterization of Pyridine–Oxazoline-Ligated Cobalt Catalysts

**Scheme 2:**
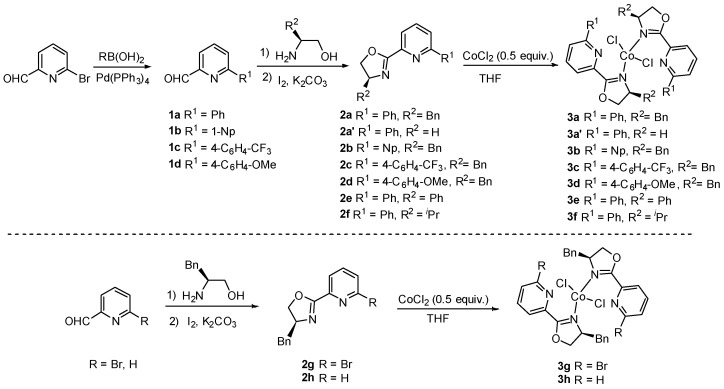
The preparation of pyridine–oxazoline-ligated cobalt complexes.

**Figure 2:**
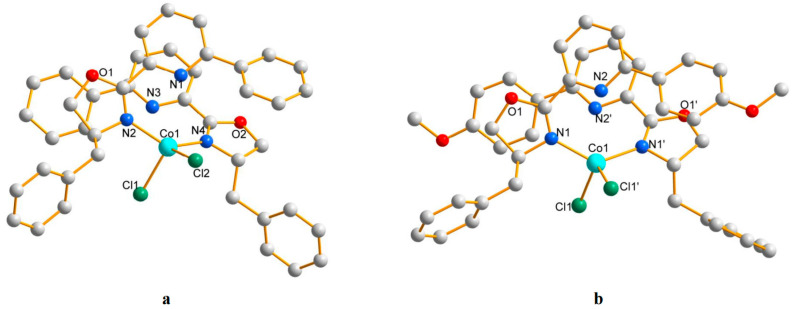
(**a**) Molecular structure of **3a** (CCDC number 2257610): selected bond lengths (Å) and angles (^o^): Co1-N2 2.036(4), Co1-N4 2.041(4), Co1-Cl1 2.2912(14), Co1-Cl2 2.2771(14), N2-Co1-Cl1 98.15(12), N2-Co1-Cl2 106.64(12), N2-Co1-N4 135.31(16), N4-Co1-Cl1 103.29(11), N4-Co1-Cl2 103.28(11). (**b**) Molecular structure of **3d** (CCDC number 2257612): selected bond lengths (Å) and angles (^o^): Co1-N1 2.025(3), Co1-N1′ 2.024(3), Co1-Cl1 2.2710(9), Cl1′-Co1-Cl1 106.53(5), N1-Co1-Cl1 101.50(8), N1-Co1-Cl1′ 106.60(8), N1′-Co1-N1 132.16(14). H atoms are omitted for clarity. Ellipsoids set at 50% probability.

The synthetic route for pyridine–oxazoline-ligated cobalt complexes is shown in Scheme 2. These cobalt complexes were fully characterized through elemental analysis, FT-IR, and high-resolution mass spectrometry. By comparing the FT-IR of ligands **2a**–**h** (1636~1644 cm^−1^) with the cobalt complexes **3a**–**h** (1586~1594 cm^−1^), it is apparent that the oxazoline C=N absorption bands of the cobalt complexes are red-shifted (SI, Figure S59), which can prove that there is an obvious coordination effect between the ligand and the cobalt center. The structure of the complexes **3a** and **3d** were further verified with single-crystal X-ray diffraction according to the slow diffusion of ether into a saturated dichloromethane solution containing the cobalt catalysts. Crystal data and structure refinement for complexes **3a** and **3d** are listed in Table S1. The structures of the complexes **3a** and **3d** are shown in Figure 2. The complexes **3a** and **3d** adopted similar structures, in which one cobalt atom was coordinated with two ligands. However, the N atom of pyridine does not coordinate with the cobalt center, which is inconsistent with previous reports [40,46] and the reason for this needs to be investigated further. As shown in Figure 2, the cobalt atom is coordinated with two N atoms of two oxazoline rings, respectively, and the complexes can be described as a distorted tetrahedron configuration. The Co1-N2 and Co1-N4 bond lengths of **3a** are 2.036(4) Å and 2.041(4) Å, whereas the Co1-N1 and Co1-N1′ of **3d** are 2.025(3) Å and 2.024(3) Å, which indicated that the N atom in the oxazoline of **3d** had a stronger coordination ability to the cobalt center atom than that of the complex **3a**.

### 3.2. Isoprene Polymerization Studies

**Figure 3:**
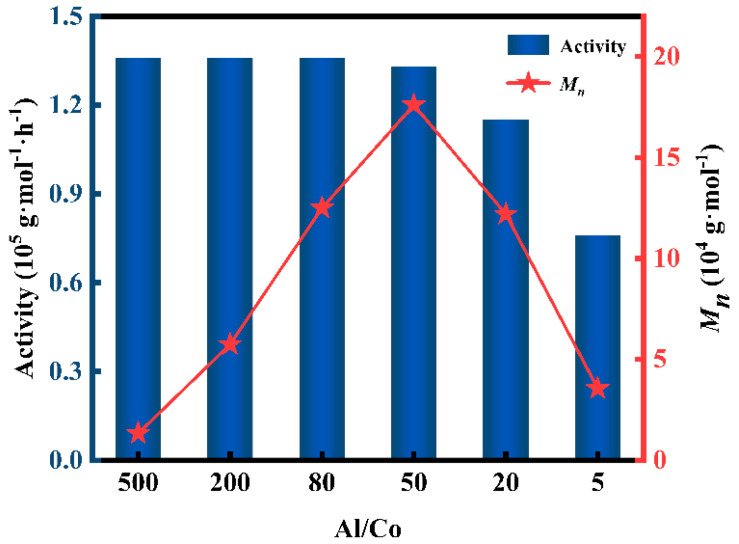
The effect of the Al/Co molar ratio on isoprene polymerization.

Initially, we chose complex **3a** as the representative pre-catalyst to catalyze the polymerization of isoprene by using different kinds of co-catalysts, including MAO, AlEt_2_Cl, trimethylaluminum (AlMe_3_), triethylaluminum (AlEt_3_) and triisobutylaluminum (Al*^i^*Bu_3_), and the resulting polymerization data are all exhibited in Table 1 (entries 1–5). It was apparent that the complex **3a** was activated for the isoprene polymerization only in the presence of AlEt_2_Cl. Based on this, we further explored the effect of the amount of AlEt_2_Cl as the co-catalyst on the isoprene polymerization (Table 1, entries 5–10). The amount of co-catalyst has a crucial effect on the activity of the catalyst and the molecular weight of the resulting polyisoprene. With the decrease in the amount of Al/Co, the catalytic activity was also decreased, while the molecular weight of the polyisoprene was increased first and then decreased (Figure 3). When the Al/Co ratio was 500, the isoprene monomer could achieve almost full conversion. With the Al/Co ratio decreasing from 500 to 50, we discovered that the catalytic activity of **3a** slightly decreased from 1.36 × 10^5^ g·mol^−1^·h^−1^ to 1.33 × 10^5^ g·mol^−1^·h^−1^, but the molecular weight increased from 1.33 × 10^4^ g·mol^−1^ to 1.76 × 10^5^ g·mol^−1^. However, the catalytic activity decreased sharply when Al/Co = 5, the monomer conversion was only 56% in two hours, and the molecular weight of the polymer was lower, at 3.56 × 10^4^ g·mol^−1^. These results may be ascribed to the fact that chain transfer to the aluminum occurs more readily in the presence of an excess of Al reagents. However, the percentage *cis*-1,4/3,4 remained almost the same, and the structure of polyisoprene basically remained, with *cis*-1,4/3,4 ≈ 2/1. The percentage of *cis*-1,4 and 3,4 units was calculated from the ^1^H NMR (see Figure S29). The effect of monomer contents on polymerization was further investigated (Table 1, entries 11–12). The conversion of isoprene was slightly decreased (from 99% to 94%) when decreasing [IP]/[Co] from 4000 to 2000, while the conversion was sharply decreased to 59% when increasing [IP]/[Co] to 8000, since the formed active center was wrapped in superabundant monomers.

**Figure 4:**
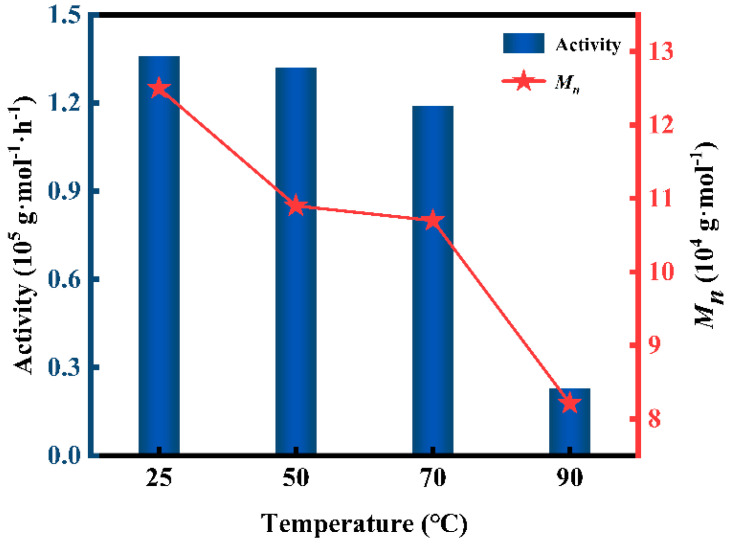
The effect of temperature on isoprene polymerization.

Subsequently, the effects of the reaction time and temperature on the polymerization of isoprene were also tested. With the reaction time decreasing from 120 min to 5 min, the conversion decreased from 99% to 76%, while the catalytic activity increased from 1.36 × 10^5^ g·mol^−1^·h^−1^ to 2.5 × 10^6^ g·mol^−1^·h^−1^ (Table 2, entries 1–5), which indicated that the active species of complex **3a** was formed rapidly and promoted the rate of isoprene polymerization. Obviously, the molecular weight was decreased with the shortening of the reaction time, but the *cis*-1,4 selectivity remained almost similar. The reaction temperature played a key role in polymerization, and when the temperature was elevated from 25 °C to 90 °C, it showed that the conversion of the isoprene monomers was decreased (Table 2 and Figure 4). For example, the catalytic activity at 70 °C was slightly decreased compared to the activity at room temperature (1.19 × 10^5^ g·mol^−1^·h^−1^ vs. 1.36 × 10^5^ g·mol^−1^·h^−1^). However, the conversion decreased sharply to 16% when the temperature increased to 90 °C, because the formed active species were unstable at a higher temperature. It was notable that the molecular weight declined from 12.5 × 10^4^ g·mol^−1^ to 8.21 × 10^4^ g·mol^−1^, and Ð increased from 2.05 to 2.49 with the elevated temperature, which was probably related to the fast chain transfer reaction at the increased temperature.

To further explore the steric and electronic effects on catalytic performance, the complexes **3a**–**h** were tested in polymerization and the resultant data were concluded in Table 3. All complexes almost appeared to exhibit high catalytic activity (1.31~1.36 × 10^5^ g·mol^−1^·h^−1^) in isoprene polymerization and obtained high-molecular-weight polyisoprene (up to 1.44 × 10^5^ g·mol^−1^, Figure S52), whereas the *cis*-1,4 selectivity almost remained unchanged (*cis*-1,4-unit contents: 62~67%). Among the complexes, complex **3a** displayed the higher catalytic activity (1.36 × 10^5^ g·mol^−1^·h^−1^) with a higher molecular weight (1.25 × 10^5^ g·mol^−1^, Figure S46) and the lowest Ð (2.05). Additionally, achiral complex **3a′** was also synthesized in order to make a comparison with chiral cobalt complex **3a** (Table 3, entry 2). The catalytic activity of **3a′** slightly decreased (1.32 × 10^5^ g·mol^−1^ ·h^−1^ vs. 1.36 × 10^5^ g·mol^−1^ ·h^−1^) and the ratio *cis*-1,4/3,4 was almost the same. However, the molecular weight of the polymer was decreased (12.5 × 10^4^ g·mol^−1^ vs. 3.15 × 10^4^ g·mol^−1^, Figure S46 vs. Figure S51) and the Ð became broader (2.05 vs. 2.64).

## 4. Conclusions

In conclusion, a family of well-defined pyridine–oxazoline cobalt complexes were synthesized and characterized, and single-crystal X-ray analysis displayed that the complexes **3a** and **3d** adopted a distorted tetrahedron configuration. In the presence of AlEt_2_Cl as a co-catalyst, these cobalt complexes all exhibited high activity for isoprene polymerization. The resultant polymers had high molecular weights (up to 1.44 × 10^5^ g·mol^−1^) and moderate *cis*-1,4 selectivity. In particular, the complex **3a** still displayed a high activity even at a relatively high polymerization temperature of 70 °C, which revealed that the formed active species of **3a** exhibited better thermal stability.

## Figures and Tables

**Table 1:** Optimization of co-catalysts for isoprene polymerization catalyzed by **3a**. ^a^

Entry	Co-Catalyst	Al/Co	Conv/%	Activity ^b^	Microstructure ^c^		*M_n_* ^d^	Ð ^d^
					*cis*-1,4	3,4		
1	MAO	500	trace	-	-	-	-	-
2	AlMe_3_	500	-	-	-	-	-	-
3	AlEt_3_	500	-	-	-	-	-	-
4	AliBu_3_	500	-	-	-	-	-	-
5	AlEt_2_Cl	500	99	1.36	67	33	1.33	2.81
6	AlEt_2_Cl	200	99	1.36	65	35	5.74	2.45
7	AlEt_2_Cl	80	99	1.36	64	36	12.5	2.05
8	AlEt_2_Cl	50	97	1.33	67	33	17.6	2.35
9	AlEt_2_Cl	20	84	1.15	62	38	12.2	2.03
10	AlEt_2_Cl	5	56	0.76	65	35	3.56	2.56
11 ^e^	AlEt_2_Cl	80	94	0.64	63	37	10.5	2.82
12 ^f^	AlEt_2_Cl	80	59	1.61	63	37	11.5	1.95

^a^ General conditions: isoprene 2 mL, 4000 equiv.; Co(Ⅱ) complex 5 μmol, 1 equiv.; toluene 5 mL; reaction temperature 25 °C; reaction time 2 h. ^b^ 10^5^ g·mol^−1^·h^−1^. ^c^ Determined by ^1^H/^13^C NMR in CDCl_3_. ^d^ *M_n_*: 10^4^ g·mol^−1^, determined by GPC. ^e^ Isoprene 1 mL, 2000 equiv. ^f^ Isoprene 4 mL, 8000 equiv.

**Table 2:** Isoprene polymerization catalyzed by **3a** under various conditions. ^a^

Entry	T/°C	t/min	Conv/%	Activity ^b^	Microstructure ^c^		*M_n_* ^d^	Ð ^d^
					*cis*-1,4	3,4		
1	25	120	99	1.36	64	36	12.5	2.05
2	25	60	97	2.64	63	37	8.79	1.95
3	25	30	94	5.12	64	36	7.67	2.79
4	25	10	83	13.6	63	37	4.46	1.79
5	25	5	76	25.0	63	37	0.47	2.61
6	50	120	92	1.32	65	35	10.9	2.21
7	70	120	82	1.19	65	35	10.7	2.49
8	90	120	16	0.23	66	34	8.21	2.30

^a^ General conditions: isoprene 2 mL, 4000 equiv.; Co(Ⅱ) complex 5 μmol, 1 equiv.; Al/Co = 80; toluene 5 mL. ^b^ 10^5^ g·mol^−1^·h^−1^. ^c^ Determined by ^1^H/^13^C NMR in CDCl_3_. ^d^ *Mn*: 10^4^ g·mol^−1^, determined by GPC.

**Table 3:** Isoprene polymerization catalyzed by Co(Ⅱ) complexes ^a^.

Entry	Cat	Conv/%	Activity ^b^	Microstructure ^c^		*M_n_* ^d^	Ð ^d^
				*cis*-1,4	3,4		
1	**3a**	99	1.36	64	36	12.5	2.05
2	**3a′**	97	1.32	64	36	3.15	2.64
3	**3b**	99	1.36	65	35	14.4	2.14
4	**3c**	99	1.36	65	35	1.92	2.75
5	**3d**	99	1.35	65	35	7.74	2.18
6	**3e**	98	1.32	65	35	4.66	2.65
7	**3f**	99	1.34	65	35	5.78	2.60
8	**3g**	96	1.31	62	38	1.70	3.42
9	**3h**	99	1.36	64	36	10.5	2.07

^a^ General conditions: isoprene 2 mL, 4000 equiv.; Co(Ⅱ) complex 5 μmol, 1 equiv.; Al/Co = 80; toluene 5 mL; reaction temperature 25 °C; reaction time 2 h. ^b^ 10^5^ g·mol^−1^·h^−1^. ^c^ Determined by ^1^H/^13^C NMR in CDCl_3_. ^d^ *M_n_*: 10^4^ g·mol^−1^, determined by GPC.

## Data Availability

Data are contained within the article and Supplementary Materials.

## Supplementary Materials

The following supporting information can be downloaded at: https://www.mdpi.com/article/10.3390/polym16050578/s1.

## References

[1] Li S.-H., Cui D.-M., Li D.-F., Hou Z.-M. (2009). Highly 3,4-Selective Polymerization of Isoprene with NPN Ligand Stabilized Rare-Earth Metal Bis(alkyl)s. Structures and Performances. Organometallics.

[2] Wang X.-X., Fan L.-L., Huang C.-B., Tong T.-L., Guo C.-Y., Sun W.-H. (2016). Highly *Cis*-1,4 Selective Polymerization of Isoprene Promoted by α-Diimine Cobalt(II) Chlorides. J. Polym. Sci. Part A Polym. Chem..

[3] Zhao J.-Y., Chen H.-F., Li W.-X., Jia X.-Y., Zhang X.-Q., Gong D.-R. (2018). Polymerization of Isoprene Promoted by Aminophosphine(ory)-Fused Bipyridine Cobalt Complexes: Precise Control of Molecular Weight and *Cis*-1,4-*alt*-3,4 Sequence. Inorg. Chem..

[4] Ricci G., Pampaloni G., Sommazzi A., Masi F. (2021). Dienes polymerization: Where we are and what lies ahead. Macromolecules.

[5] Ricci G., Sommazzi A., Masi F., Ricci M., Boglia A., Leone G. (2010). Well-defined transition metal complexes with phosphorus and nitrogen ligands for 1,3-dienes polymerization. Coord. Chem. Rev..

[6] Song J.S., Huang B.C., Yu D.S. (2001). Progress of synthesis and application of trans-1,4-polyisoprene. J. App. Polym. Sci..

[7] Thiele S.K.-H., Wilson D.R. (2003). Alternate Transition Metal Complex Based Diene Polymerization. J. Macromol. Sci. Polym. Rev..

[8] Halasa A.F., Hsu W.L. (2002). Synthesis of High Vinyl Elastomers via Mixed Organolithium and Sodium Alkoxide in the Presence of Polar Modifier. Polymer.

[9] Yang Y., Liu B., Lv K., Gao W., Cui D.-M., Chen X.-S., Jing X.-B. (2007). Pyrrolide-Supported Lanthanide Alkyl Complexes. Influence of Ligands on Molecular Structure and Catalytic Activity toward Isoprene Polymerization. Organometallics.

[10] Zhang L.-X., Luo Y., Hou Z.-M. (2005). Unprecedented Isospecific 3,4-Polymerization of Isoprene by Cationic Rare Earth Metal Alkyl Species Resulting from a Binuclear Precursor. J. Am. Chem. Soc..

[11] Wang B., Cui D., Lv K. (2008). Highly 3,4-selective living polymerization of isoprene with rare earth metal fluorenyl N-heterocyclic carbene precursors. Macromolecules.

[12] Liu H., He J., Liu Z., Lin Z., Du G., Zhang S., Li X. (2013). Quasi-living trans-1,4-polymerization of isoprene by cationic rare earth metal alkyl species bearing a chiral (S,S)-bis(oxazolinylphenyl)amido ligand. Macromolecules.

[13] Dai Q.-Q., Jia X.-Y., Yang F., Bai C.-X., Hu Y.-M., Zhang X.-Q. (2016). Iminopyridine-Based Cobalt(II) and Nickel(II) Complexes: Synthesis, Characterization, and Their Catalytic Behaviors for 1,3-Butadiene Polymerization. Polymers.

[14] Xiao T.P.-F., Zhang S., Kehr G., Hao X., Erker G., Sun W.-H. (2011). Bidentate Iron(II) Dichloride Complexes Bearing Substituted 8-(Benzimidazol-2-yl)quinolines: Synthesis, Characterization, and Ethylene Polymerization Behavior. Organometallics.

[15] Xiao T.P.-F., Zhang S., Li B.-X., Hao X., Redshaw C., Li Y.-S., Sun W.-H. (2011). Ferrous and Cobaltous Chloride Complexes Bearing 2-(1-(Arylimino)methyl)-8-(1H-benzimidazol-2-yl)quinolines: Synthesis, Characterization and Catalytic Behavior in Ethylene Polymerization. Polymer.

[16] Ashitaka H., Ishikawa H., Ueno H., Nagasaka A. (1983). Syndiotactic 1,2-Polybutadiene with Co-CS_2_ Catalyst system. I. Preparation, Properties, and Application of Highly Crystalline Syndiotactic 1,2-Polybutadiene. J. Polym. Sci. Polym. Chem. Ed..

[17] Ashitaka H., Inaishi K., Ueno H. (1983). Syndiotactic 1,2-Polybutadiene with Co-CS_2_ Catalyst System. III.^1^H- and ^13^C-NMR Study of Highly Syndiotactic 1,2-Polybutadiene. J. Polym. Sci. Polym. Chem. Ed..

[18] Racanelli P., Porri L. (1970). *Cis*-1,4-polybutadiene by Cobalt Catalysts. Some Features of the Catalysts Prepared from Alkyl Aluminium Compounds Containing Al-O-Al Bonds. Eur. Polym. J..

[19] Ricci G., Italia S., Porri L. (1988). Polymerization of Butadiene to 1,2-Syndiotactic Polymer with (η_3_-C_8_H_13_)(C_4_H_6_)Co. Some Observations on the Factors That Determine the Stereospecificity. Polym. Commun..

[20] Appukuttan V., Zhang L., Ha C.-S., Kim I. (2009). Highly Active and Stereospecific Polymerizations of 1,3-Butadiene by Using Bis(benzimidazolyl)amine Ligands Derived Co(II) Complexes in Combination with Ethylaluminum Sesquichloride. Polymer.

[21] Cai Z.-G., Shinzawa M., Nakayama Y., Shiono T. (2009). Synthesis of Regioblock Polybutadiene with CoCl_2_-Based Catalyst via Reversible Coordination of Lewis Base. Macromolecules.

[22] Gong D.-R., Wang B.-L., Bai C.-X., Bi J.-F., Wang F., Dong W.-M., Zhang X.-Q., Jiang L.-S. (2009). Metal Dependent Control of *Cis*-/*trans*-1,4 Regioselectivity in 1,3-Butadiene Polymerization Catalyzed by Transition Metal Complexes Supported by 2,6-Bis[1-(iminophenyl)ethyl]pyridine. Polymer.

[23] Gong D.-R., Wang B.-L., Cai H.-G., Zhang X.-Q., Jiang L.-S. (2011). Synthesis, Characterization and Butadiene Polymerization Studies of Cobalt(II) Complexes Bearing Bisiminopyridine Ligand. J. Organomet. Chem..

[24] Gong D.-R., Wang B.-L., Jia X.-Y., Zhang X.-Q. (2014). The Enhanced Catalytic Performance of Cobalt Catalysts towards Butadiene Polymerization by Introducing a Labile Donor in a Salen Ligand. Dalton Trans..

[25] Jie S.-Y., Ai P.-F., Li B.-G. (2011). Highly Active and Stereospecific Polymerization of 1,3-Butadiene Catalyzed by Dinuclear Cobalt(II) Complexes Bearing 3-Aryliminomethyl-2-hydroxybenzaldehydes. Dalton Trans..

[26] Nobbs J.D., Tomov A.K., Cariou R., Gibson V.C., White A.J., Britovsek G.J. (2012). Thio-Pybox and Thio-Phebox Complexes of Chromium, Iron, Cobalt and Nickel and Their Application in Ethylene and Butadiene Polymerisation Catalysis. Dalton Trans..

[27] Ricci G., Leone G., Boglia A., Bertini F., Boccia A.C., Zetta L. (2009). Synthesis and Characterization of Isotactic 1,2-Poly(*E*-3-methyl-1,3-pentadiene). Some Remarks about the Influence of Monomer Structure on Polymerization Stereoselectivity. Macromolecules.

[28] Chen X.-M., Huang L.-C., Gao W. (2021). One-pot synthesis of cobalt complexes with 2,6-bis(arylimino)phenoxyl/phenthioxyl ligands and catalysis on isoprene polymerization. Dalton Trans..

[29] You J.-Y., Chen B.-H., Gong D.-R. (2023). Polymerization of isoprene, myrcene, and butadiene catalyzed by cobalt complexes supported with 2-acetyl-6-iminopyridine ligand. Appl. Organomet. Chem..

[30] Du Y.-X., Gao S., Ma H., Lu S.-Q., Zhang Z.-H., Zhao M.-M. (2023). Catalytic Behavior of Cobalt Complexes Bearing Pyridine–Oxime Ligands in Isoprene Polymerization. Polymers.

[31] Guo L.-H., Jing X.-Y., Xiong S.-Y., Liu W.-J., Liu Y.-L., Liu Z., Chen C.-L. (2016). Influences of Alkyl and Aryl Substituents on Iminopyridine Fe(II)- and Co(II)-Catalyzed Isoprene Polymerization. Polymers.

[32] Zhu G.-Q., Zhang X.-H., Zhao M.-M., Wang L., Jing C.-Y., Wang P., Wang X.-W., Wang Q.-G. (2018). Influences of Fluorine Substituents on Iminopyridine Fe(II)- and Co(II)-Catalyzed Isoprene Polymerization. Polymers.

[33] Zhang X.-H., Zhu G.-Q., Mahmood Q., Zhao M.-M., Wang L., Jing C.-Y., Wang X.-W., Wang Q.-G. (2019). Iminoimidazole-based Co(II) and Fe(II) Complexes: Syntheses, Characterization, and Catalytic Behaviors for Isoprene Polymerization. J. Polym. Sci. Part A Polym. Chem..

[34] Zhao M.-M., Ma Y., Zhang X.-H., Wang L., Zhu G.-Q., Wang Q.-G. (2022). Synthesis, Characterization and Catalytic Property Studies for Isoprene Polymerization of Iron Complexes Bearing Unionized Pyridine-Oxime Ligands. Polymers.

[35] Fang L., Zhao W.-P., Han C., Zhang C.-Y., Liu H., Hu Y.-M., Zhang X.-Q. (2019). 1,3-Butadiene Polymerizations Catalyzed by Cobalt and Iron Dichloride Complexes Bearing Pyrazolylimine Ligands. Chin. J. Polym. Sci..

[36] Yousuf N., Ma Y.-P., Mahmood Q., Zhang W.-J., Liu M., Yuan R.-Y., Sun W.-H. (2023). Structurally Rigid (8-(Arylimino)-5,6,7-trihydroquinolin-2-yl)-methyl Acetate Cobalt Complex Catalysts for Isoprene Polymerization with High Activity and cis-1,4 Selectivity. Catalysts.

[37] Desimoni G., Faita G., Quadrelli P. (2003). Pyridine-2,6-bis(oxazolines), Helpful Ligands for Asymmetric Catalysts. Chem. Rev..

[38] Nishiyama H., Kondo M., Nakamura T., Itoh K. (1991). Highly Enantioselective Hydrosilylation of Ketones with Chiral and C_2_-symmetrical Bis(oxazolinyl)pyridine-rhodium Catalysts. Organometallics.

[39] Gao R., Xiao L.-W., Hao X., Sun W.-H., Wang F.-S. (2008). Synthesis of Benzoxazolylpyridine Nickel Complexes and Their Efficient Dimerization of Ethylene to Alpha-butene. Dalton Trans..

[40] Guo J., Liu H., Bi J.-F., Zhang C.-Y., Zhang H.-X., Bai C.-X., Hu Y.-M., Zhang X.-Q. (2015). Pyridine-oxazoline and Quinoline-oxazoline Ligated Cobalt Complexes: Synthesis, Characterization, and 1,3-Butadiene Polymerization Behaviors. Inorganica Chim. Acta.

[41] Ochędzan-Siodłak W., Bihun-Kisiel A., Siodłak D., Poliwoda A., Dziuk B. (2018). Titanium and Vanadium Catalysts with Oxazoline Ligands for Ethylene-Norbornene (co)Polymerization. Eur. Polym. J..

[42] Fu L.-R., Wang Y.-B., Jiang H., Hao X.-Q., Song M.-P. (2022). Applications of Cobalt Complexes in Olefin Polymerization. Chin. J. Org. Chem..

[43] Hou S.-Y., Hao X.-G., Jiang H., Hao X.-Q., Song M.-P. (2023). Progress in the Polymerization of 1,3-Diene Catalyzed by Fe and Co Metal Complexes. Acta Polym. Sin..

[44] Albrecht M., Lindner M.M. (2011). Cleavage of Unreactive Bonds with Pincer Metal Complexes. Dalton Trans..

[45] Wang T., Hao X.-Q., Huang J.-J., Wang K., Gong J.-F., Song M.-P. (2014). Chiral CNN Pincer Palladium(II) Complexes with 2-Aryl-6-(oxazolinyl)pyridine Ligands: Synthesis, Characterization, and Application to Enantioselective Allylation of Isatins and Suzuki-Miyaura Coupling Reaction. Organometallics.

[46] Zhao M., Zhang X., Wang L., Wang L., Zhu G., Wang Q. (2022). Pyridine-oxazoline ligated iron complexes: Synthesis, characterization, and catalytic activity for isoprene polymerization. Appl. Organomet. Chem..

