# Supplementary material for: Synthesis, Characterization, and Catalytic Behaviors in Isoprene Polymerization of Pyridine–Oxazoline-Ligated Cobalt Complexes
Source: Polymers (Basel). 2024 Feb 21;16(5):578. doi: 10.3390/polym16050578 (PMC10935341; doi:10.3390/polym16050578)
Supplement: Supplementary file 1 [file polymers-16-00578-s001.zip › polymers-2829266-supplementary.pdf]

## *Supporting information*

# Synthesis, Characterization, and Catalytic Behaviors in Isoprene Polymerization of Pyridine–Oxazoline-Ligated Cobalt Complexes

Xiuge Hao †, Jin-Kui Liu †, Weize Zhuo, Jiajing Zheng, Xin-Qi Hao, Jun-Fang Gong *, Hui Jiang * and Mao-Ping Song

College of Chemistry, Zhengzhou University, Zhengzhou 450001, China; 202012152012695@gs.zzu.edu.cn (X.H.); liujinkui28@gs.zzu.edu.cn (J.-K.L.); 202023000526@stu.zzu.edu.cn (W.Z.); zjj3412904822@stu.zzu.edu.cn (J.Z.); xqhao@zzu.edu.cn (X.-Q.H.); mpsong@zzu.edu.cn (M.-P.S.)

* Correspondence: gongjf@zzu.edu.cn (J.-F.G.); jiangh@zzu.edu.cn (H.J.)

† These authors contributed equally to this work.

### Table of Contents

1. NMR Spectra of Ligands ..... 2

2. Determination of Polymer Compositions ..... 16

3. NMR Spectra and GPC Curve of Representative Polyisoprene ..... 17

4. FT-IR Spectra of Ligands and Complexes ..... 32

5. X-ray Crystallographic Data ..... 33

## 1. NMR Spectra of Ligands

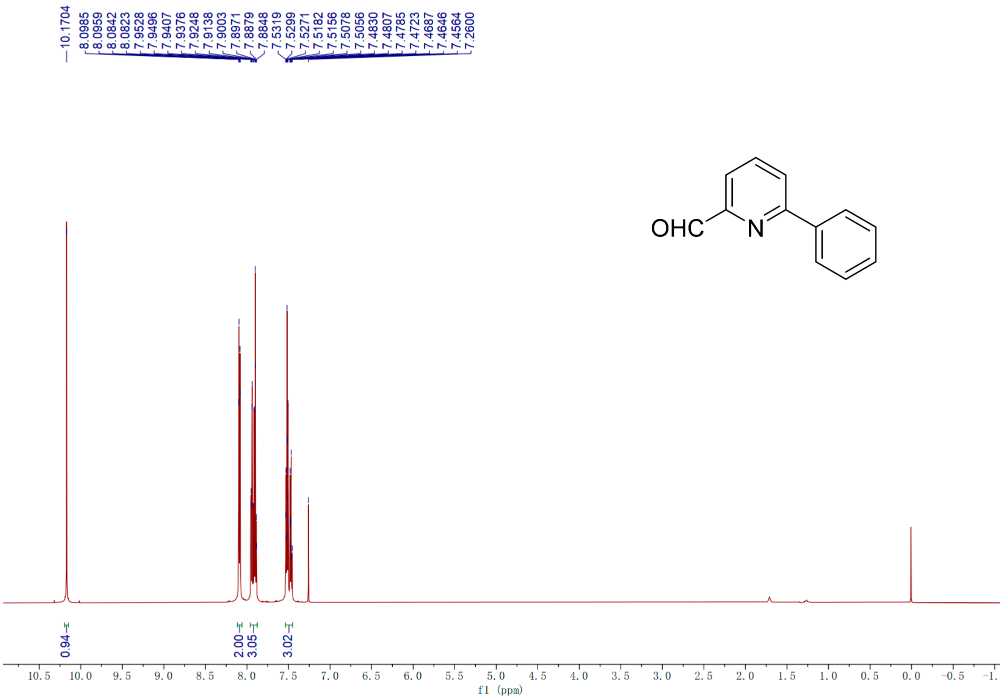


Figure **S1** $^{1}$H NMR (600 MHz, $CDCl_3$) of **1a**

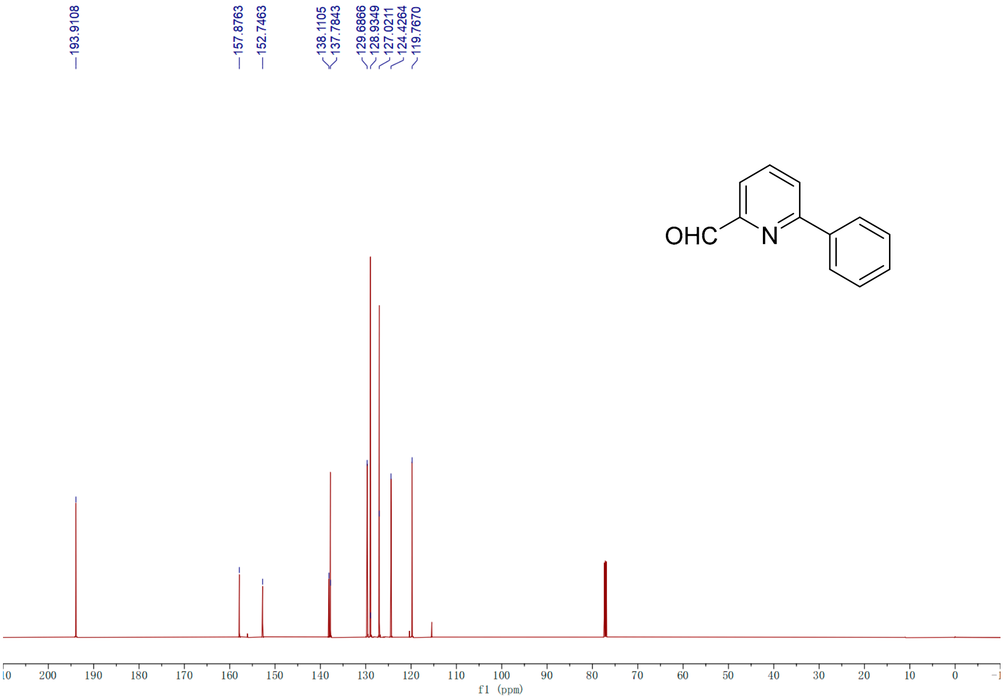


Figure **S2** $^{13}C\{^{1}H\}$ NMR (151 MHz, $CDCl_3$) of **1a**

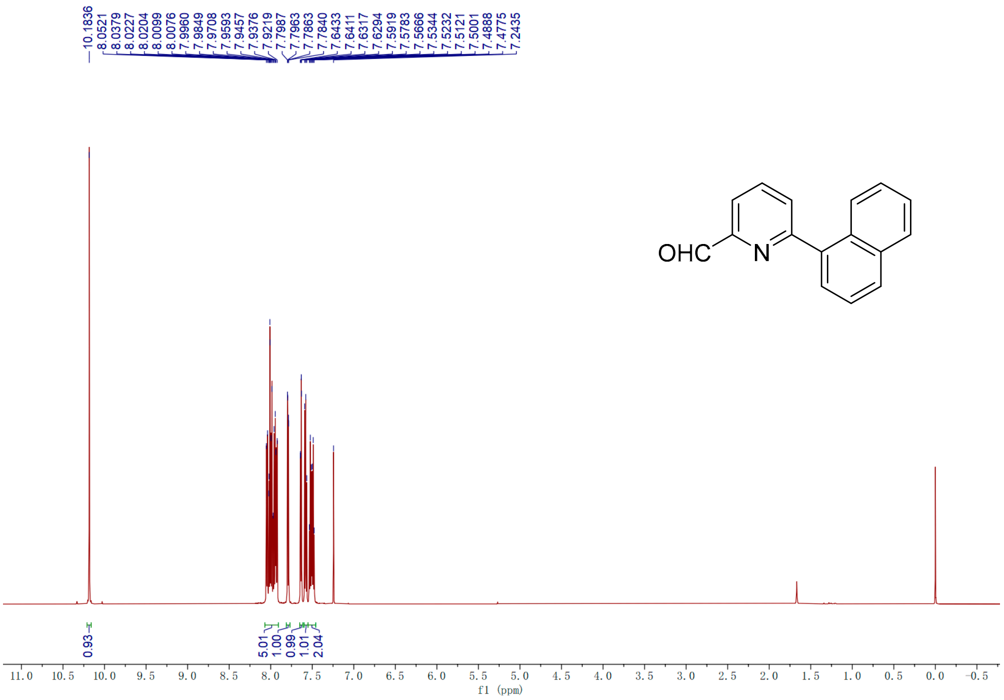

Figure **S3** $^{1}$H NMR (600 MHz, $CDCl_3$) of **1b**

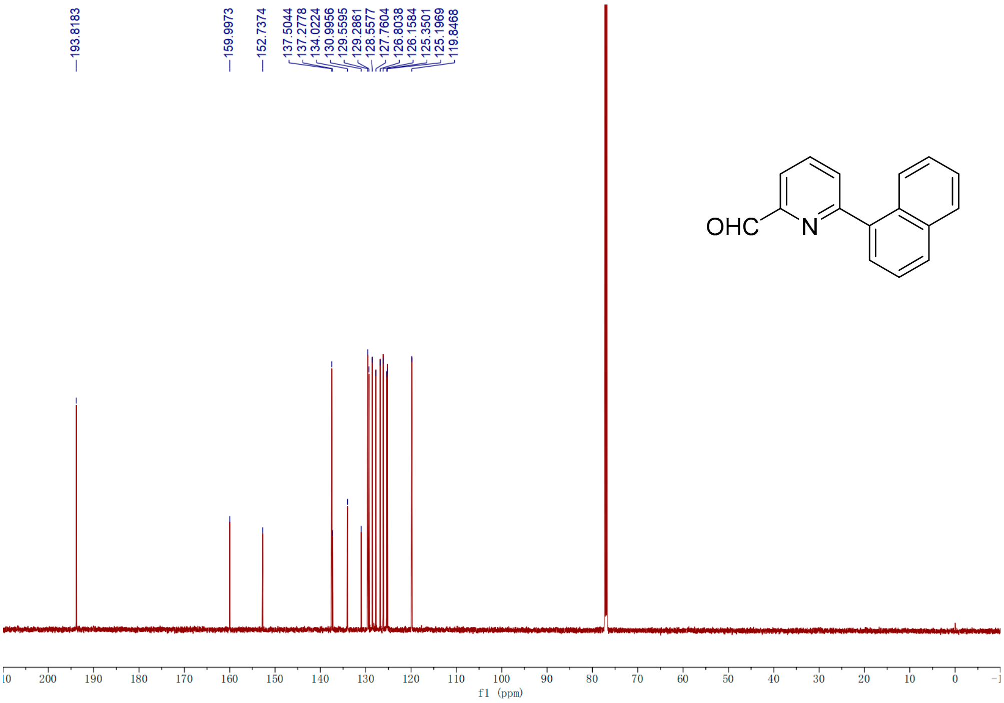

Figure **S4** $^{13}C\{^1H\}$ NMR (151 MHz, $CDCl_3$) of **1b**

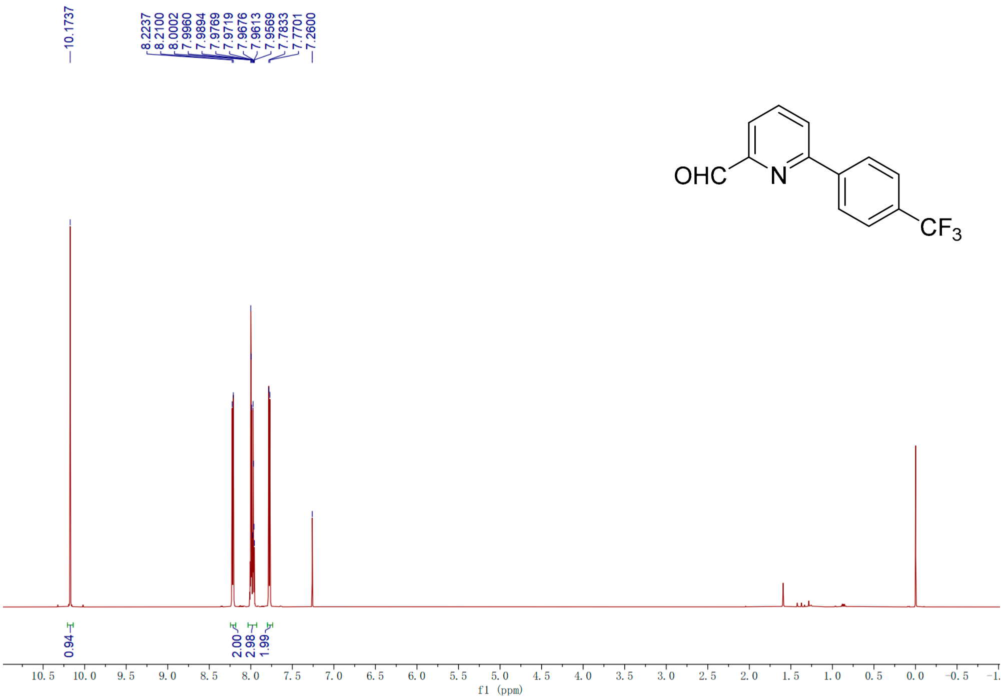

Figure **S5** $^{1}H$ NMR (600 MHz, $CDCl_3$) of **1c**

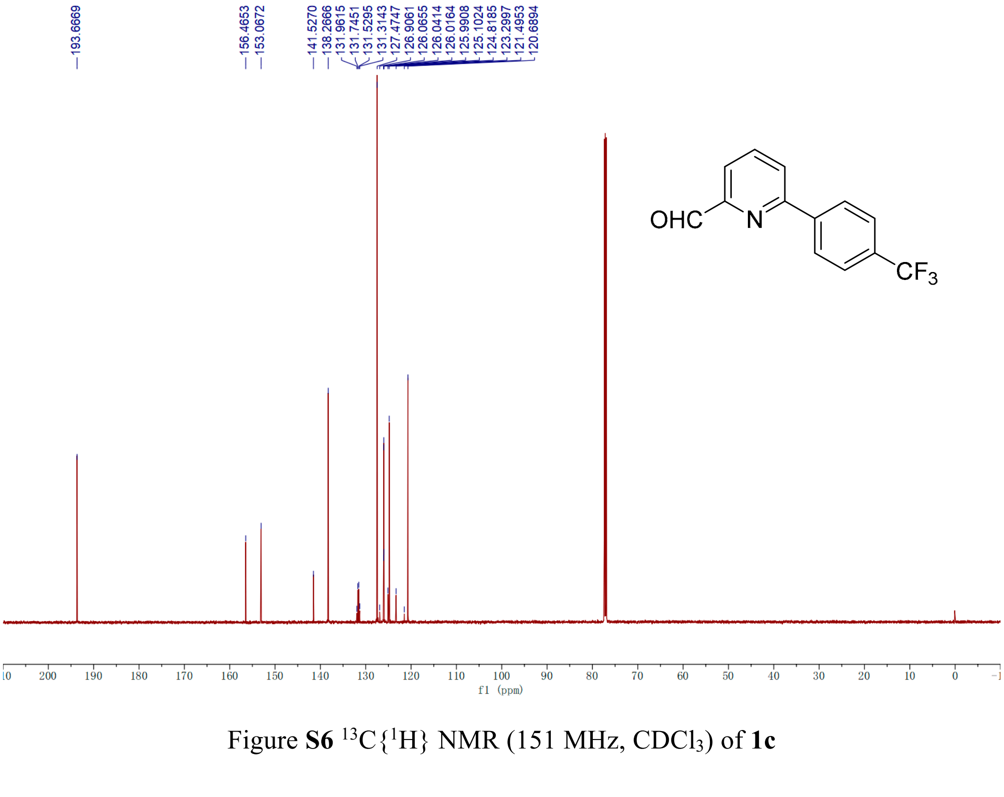

Figure **S6** $^{13}C\{^{1}H\}$ NMR (151 MHz, $CDCl_3$) of **1c**

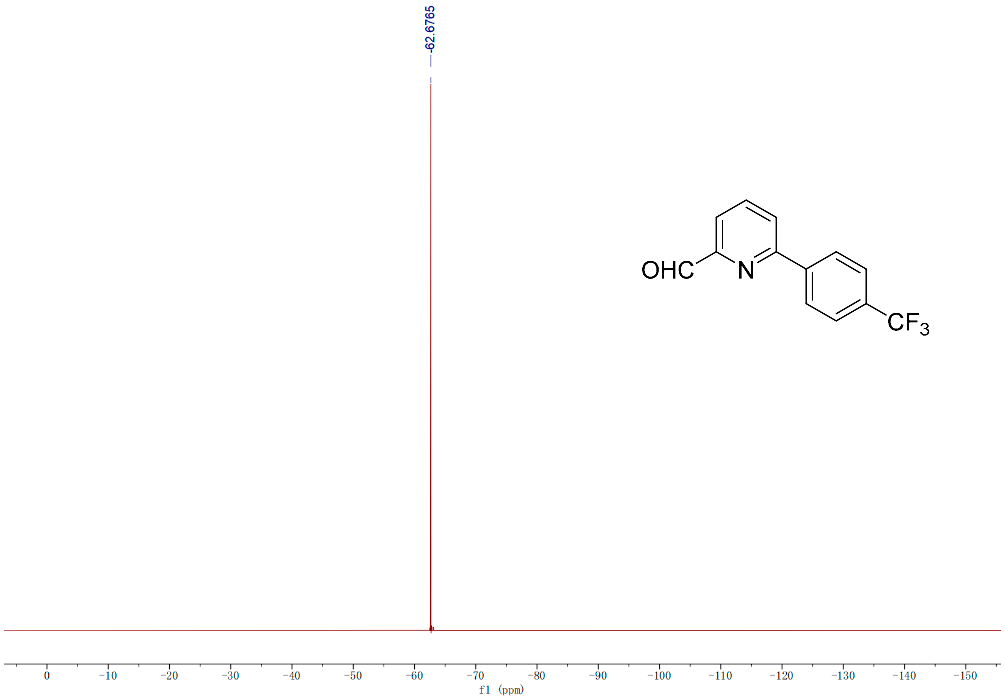


Figure **S7** $^{19}F\{^1H\}$ NMR (565 MHz, $CDCl_3$) of **1c**

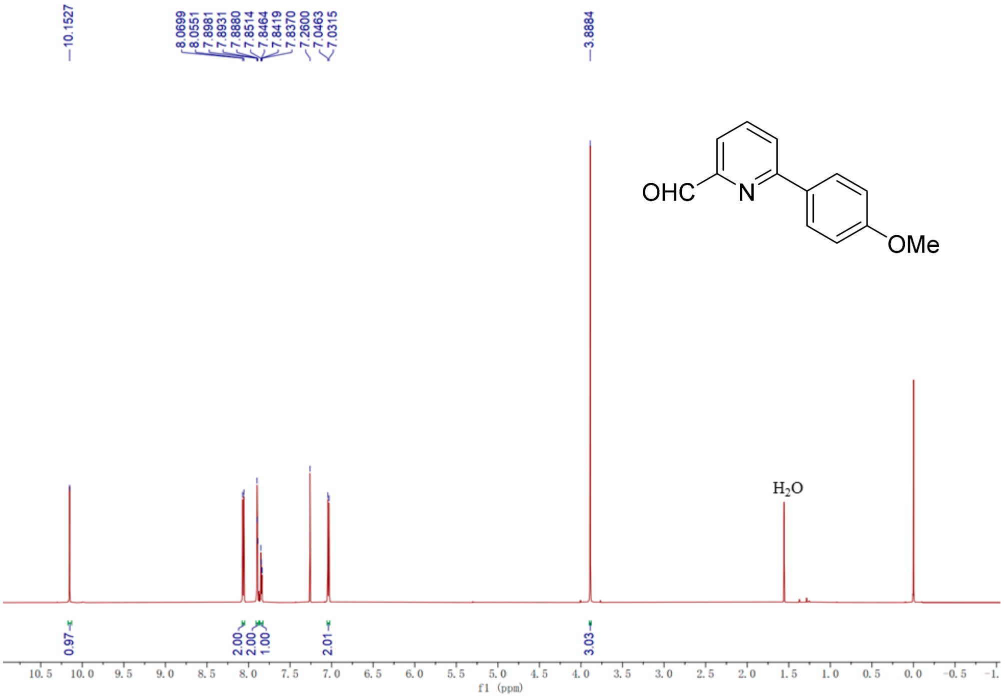


Figure **S8** $^1H$ NMR (600 MHz, $CDCl_3$) of **1d**

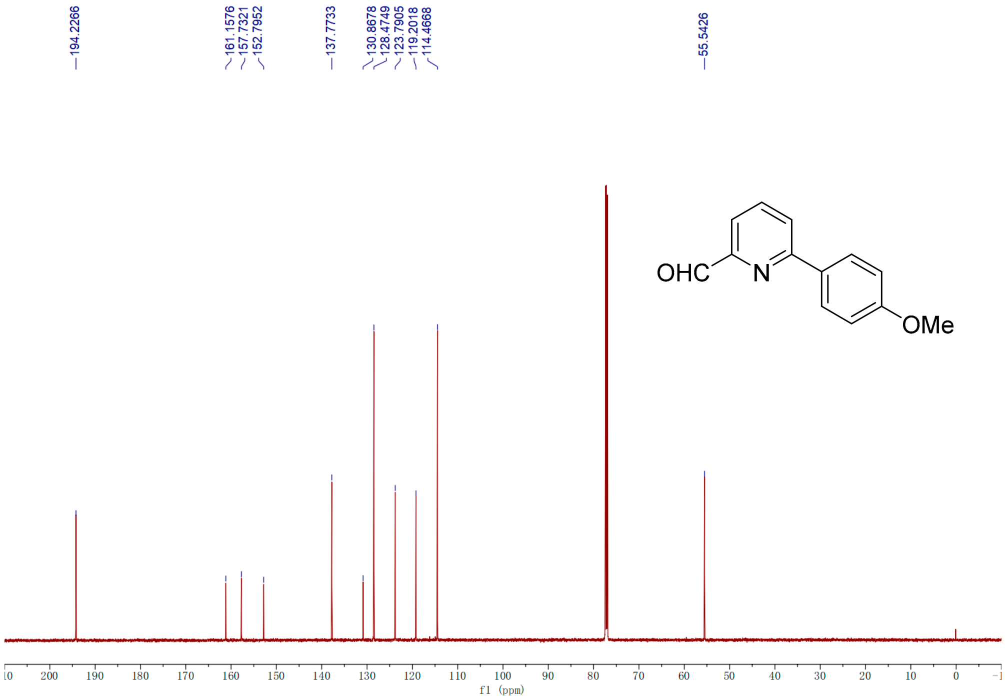

Figure **S9** $^{13}C\{^{1}H\}$ NMR (151 MHz, $CDCl_3$) of **1d**

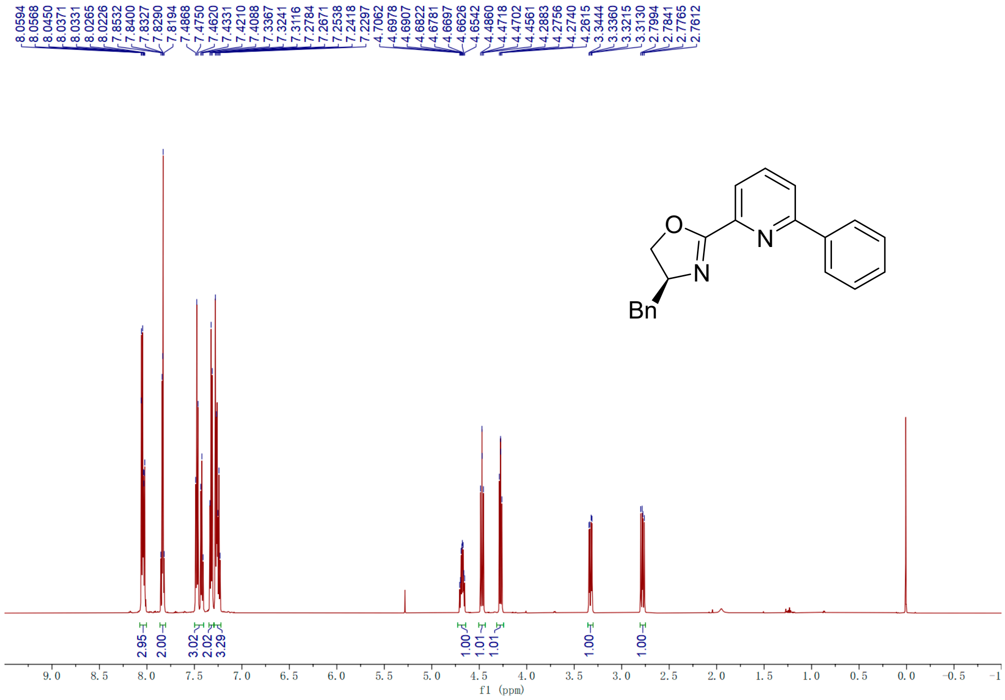

Figure **S10** $^{1}H$ NMR (600 MHz, $CDCl_3$) of **2a**

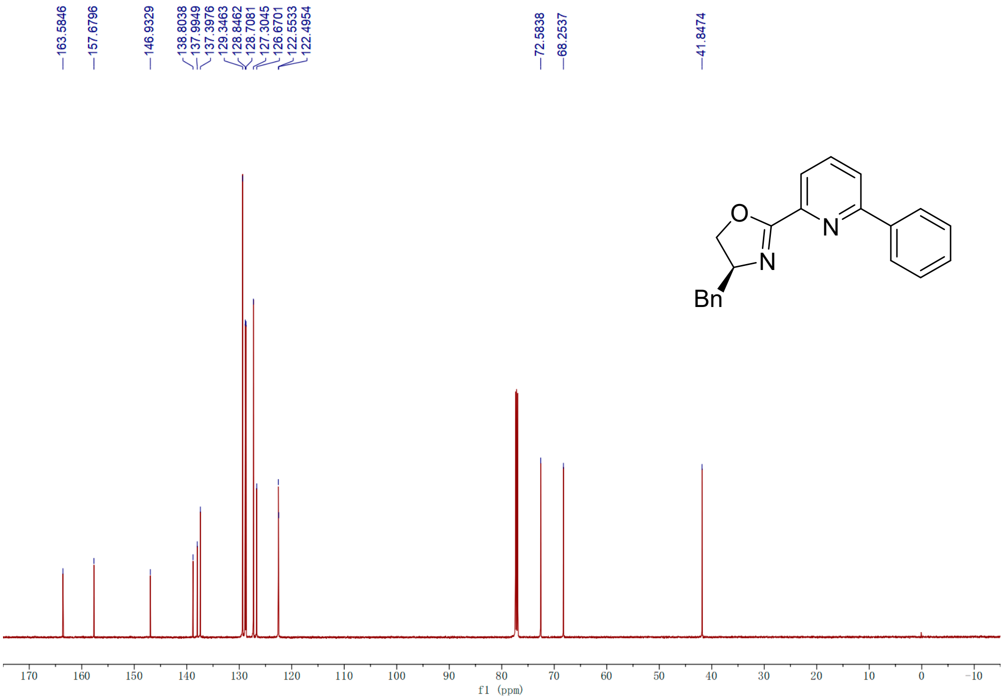


Figure **S11** $^{13}C\{^{1}H\}$ NMR (151 MHz, $CDCl_3$) of **2a**

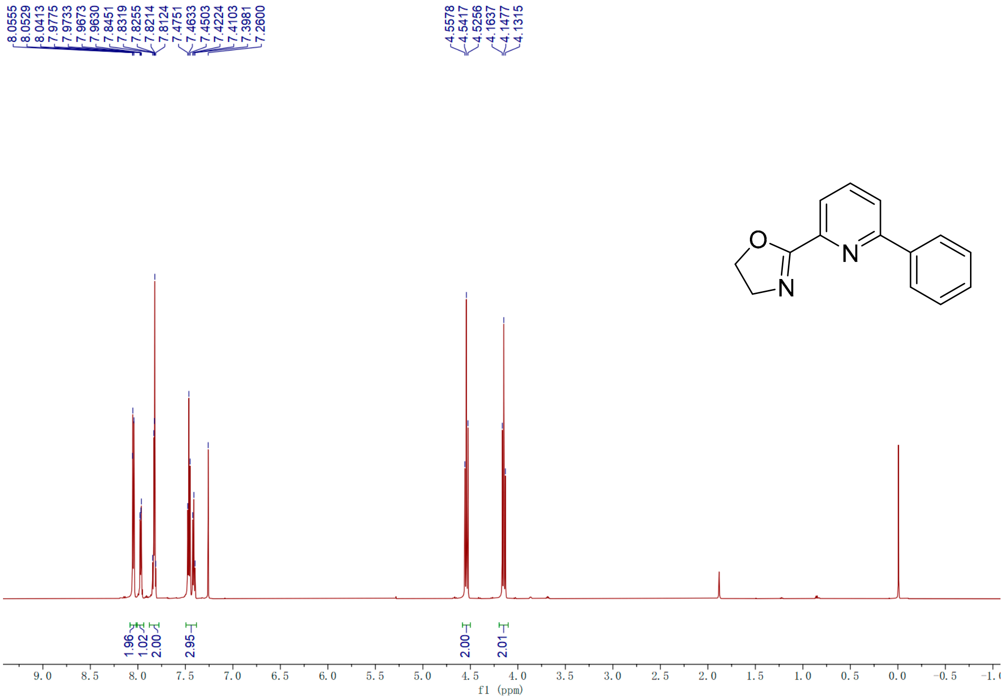


Figure **S12** $^{1}H$ NMR (600 MHz, $CDCl_3$) of **3a′**

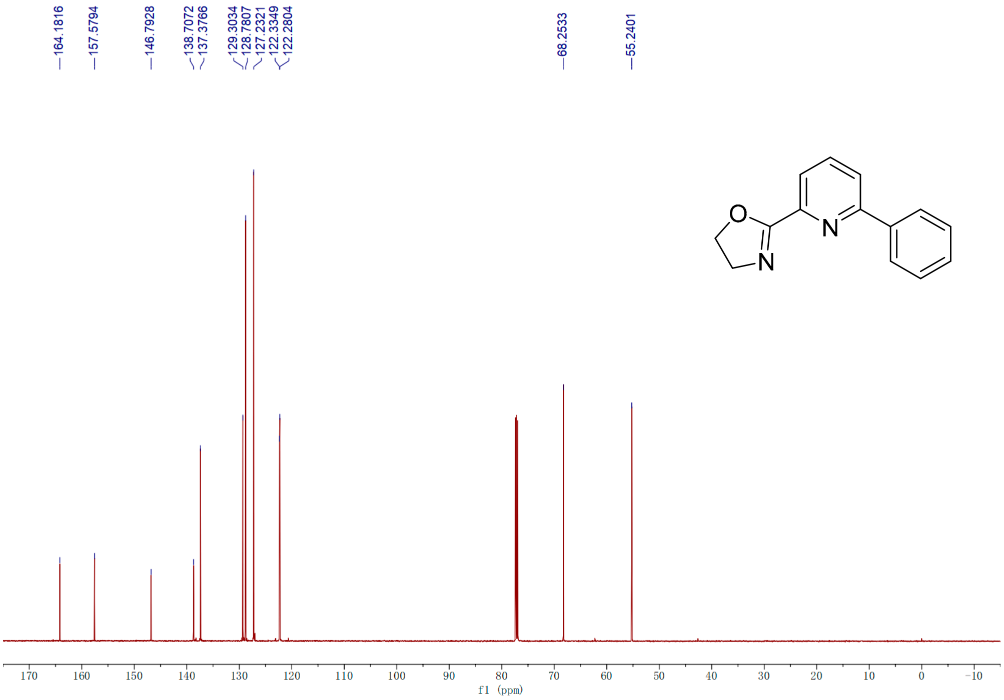

Figure **S13** $^{13}C\{^{1}H\}$ NMR (151 MHz, $CDCl_3$) of **3a′**

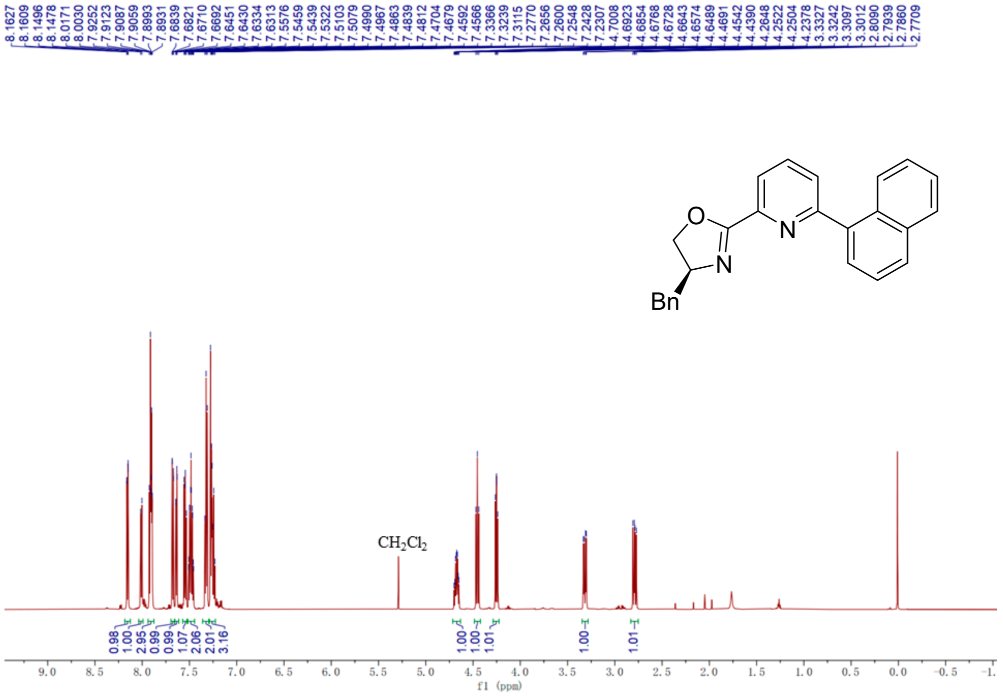

Figure **S14** $^{1}H$ NMR (600 MHz, $CDCl_3$) of **2b**

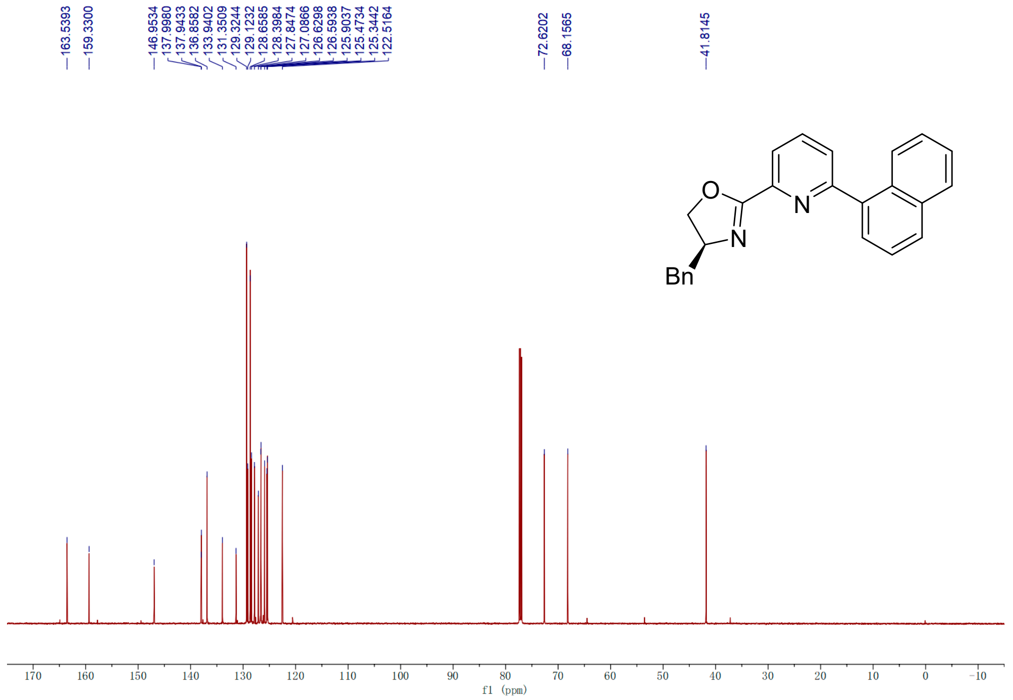

Figure **S15** $^{13}C\{^{1}H\}$ NMR (151 MHz, $CDCl_3$) of **2b**

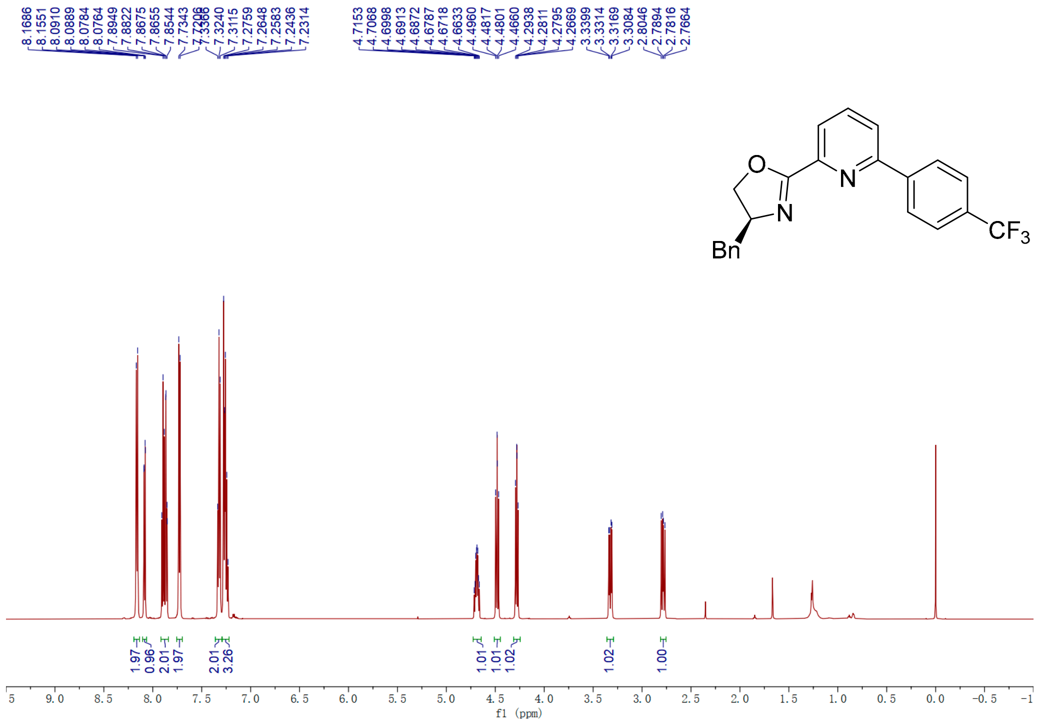

Figure **S16** $^{1}H$ NMR (600 MHz, $CDCl_3$) of **2c**

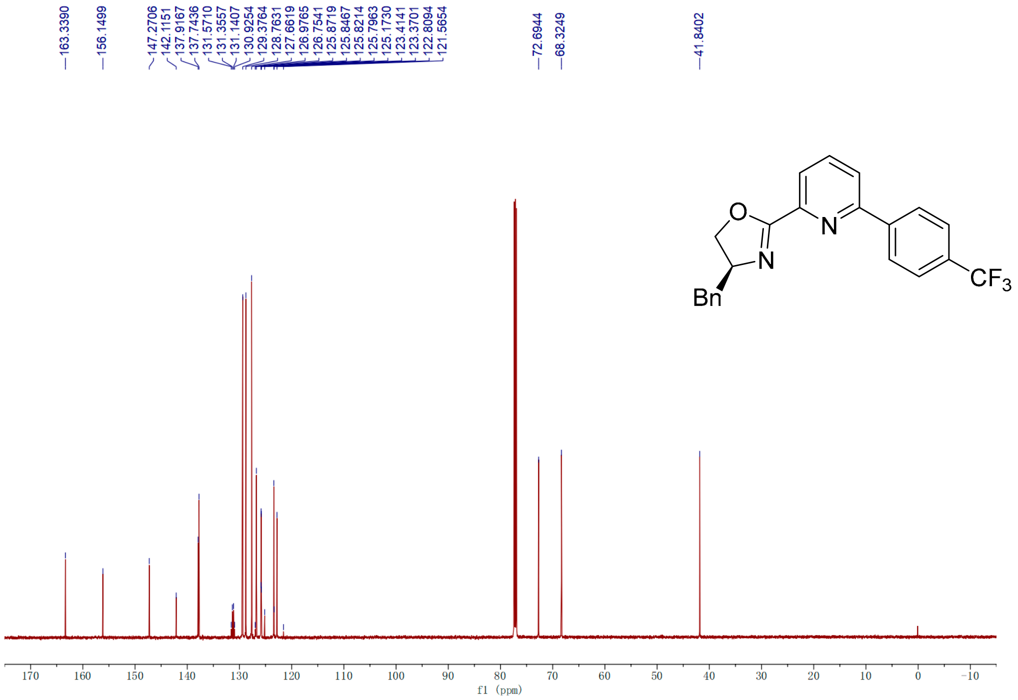

Figure **S17** $^{13}C\{^{1}H\}$ NMR (151 MHz, $CDCl_3$) of **2c**

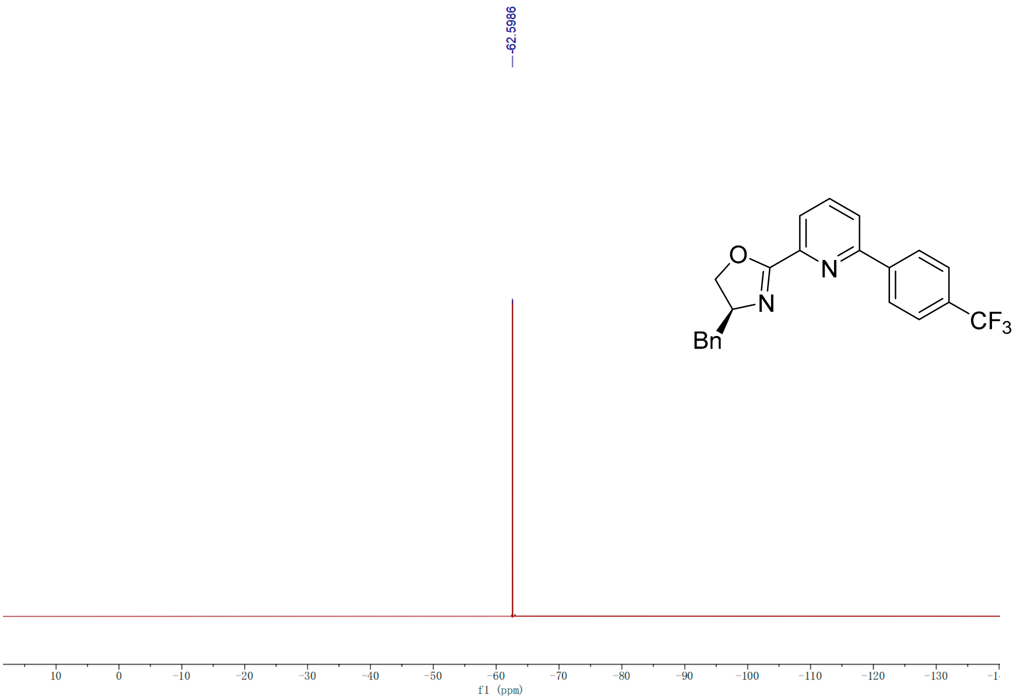

Figure **S18** $^{19}F\{^{1}H\}$ NMR (565 MHz, $CDCl_3$) of **2c**

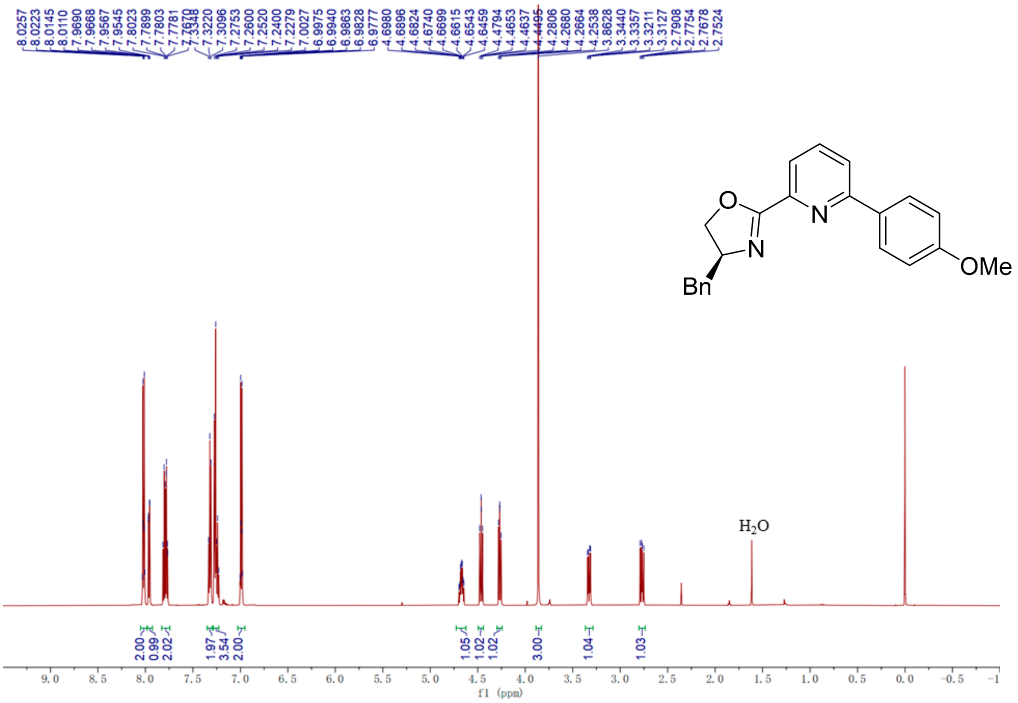

Figure **S19** $^{1}H$ NMR (600 MHz, $CDCl_3$) of **2d**

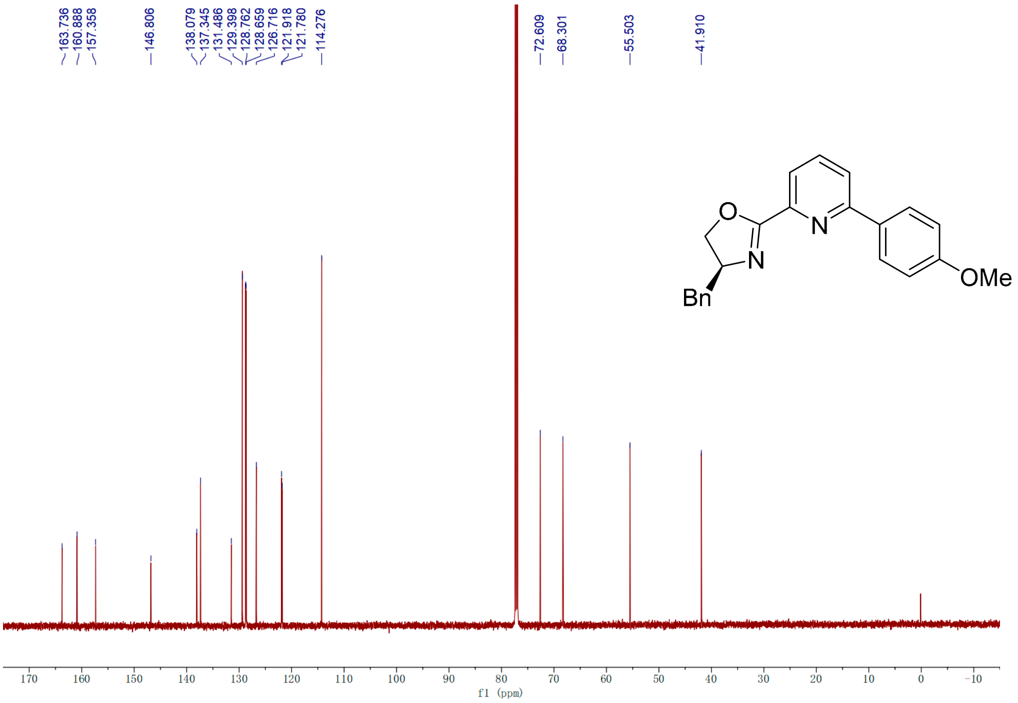

Figure **S20** $^{13}C\{^{1}H\}$ NMR (151 MHz, $CDCl_3$) of **2d**

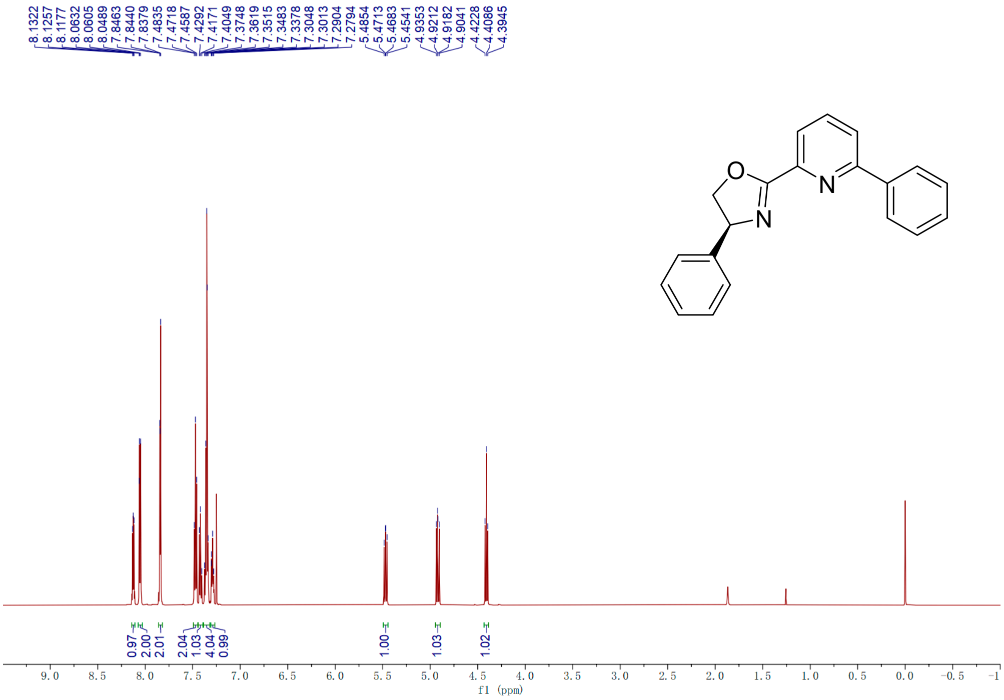


Figure **S21** $^{1}$H NMR (600 MHz, $CDCl_3$) of **2e**

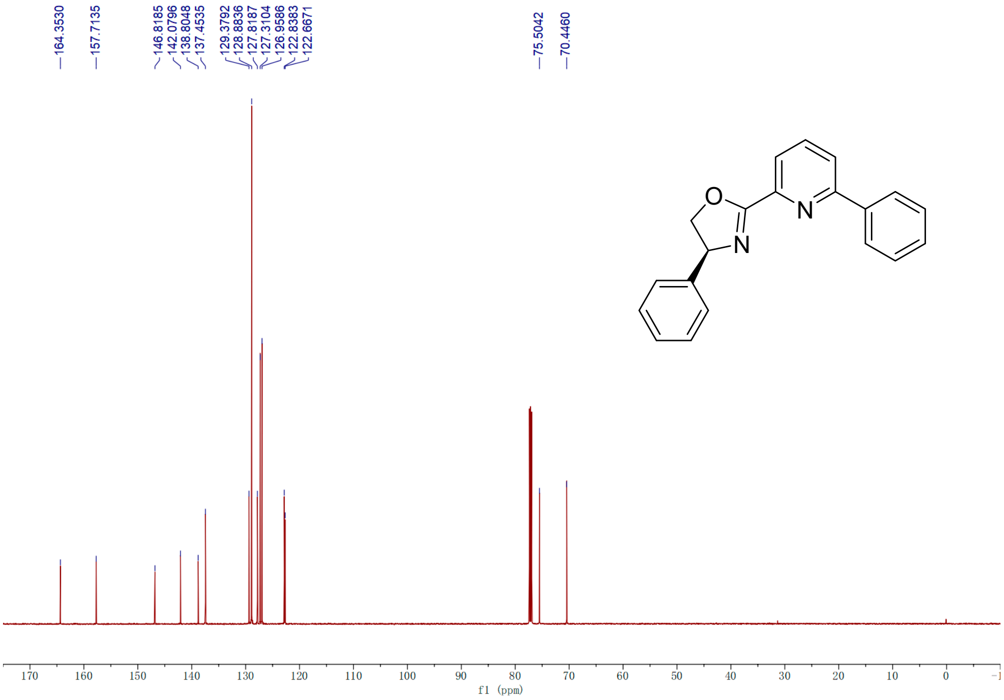


Figure **S22** $^{13}$C{$^{1}$H} NMR (151 MHz, $CDCl_3$) of **2e**

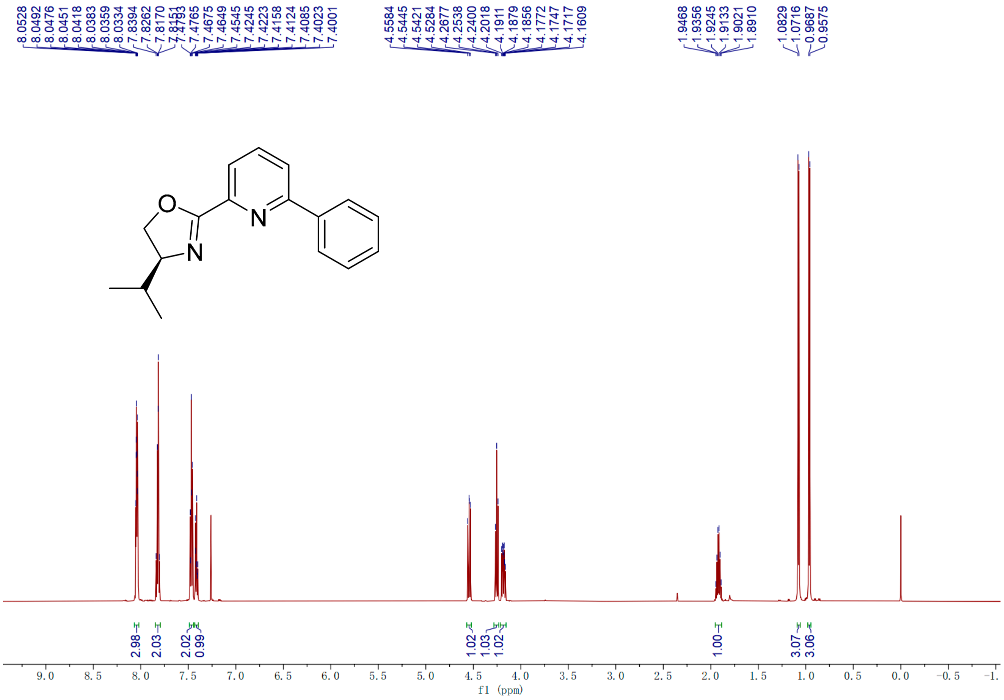

Figure **S23** $^{1}$H NMR (600 MHz, $CDCl_3$) of **2f**

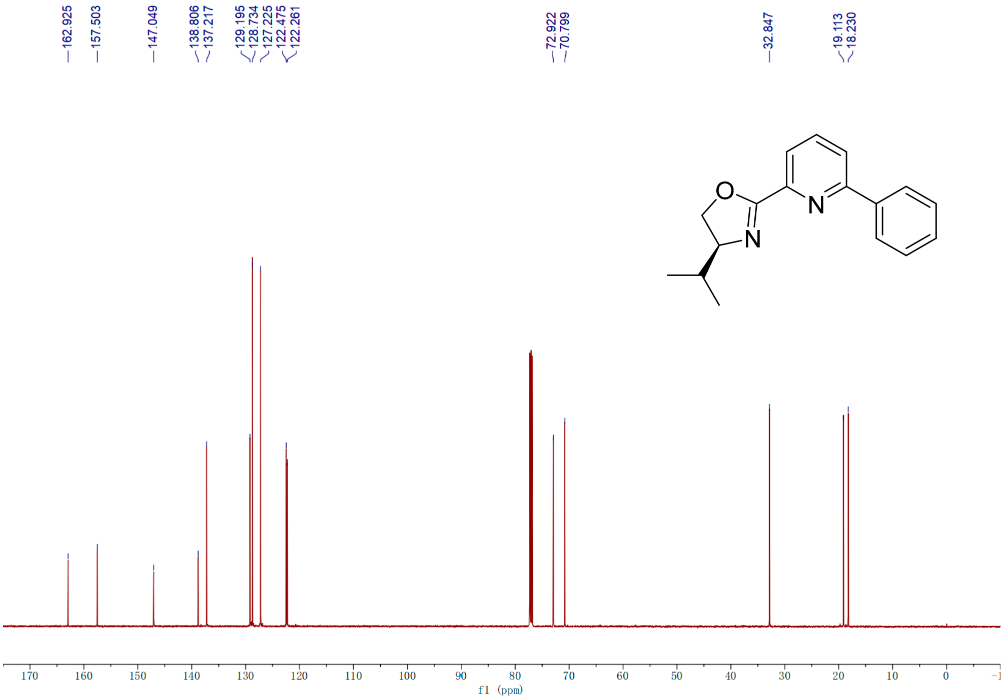

Figure **S24** $^{13}$C{$^{1}$H} NMR (151 MHz, $CDCl_3$) of **2f**

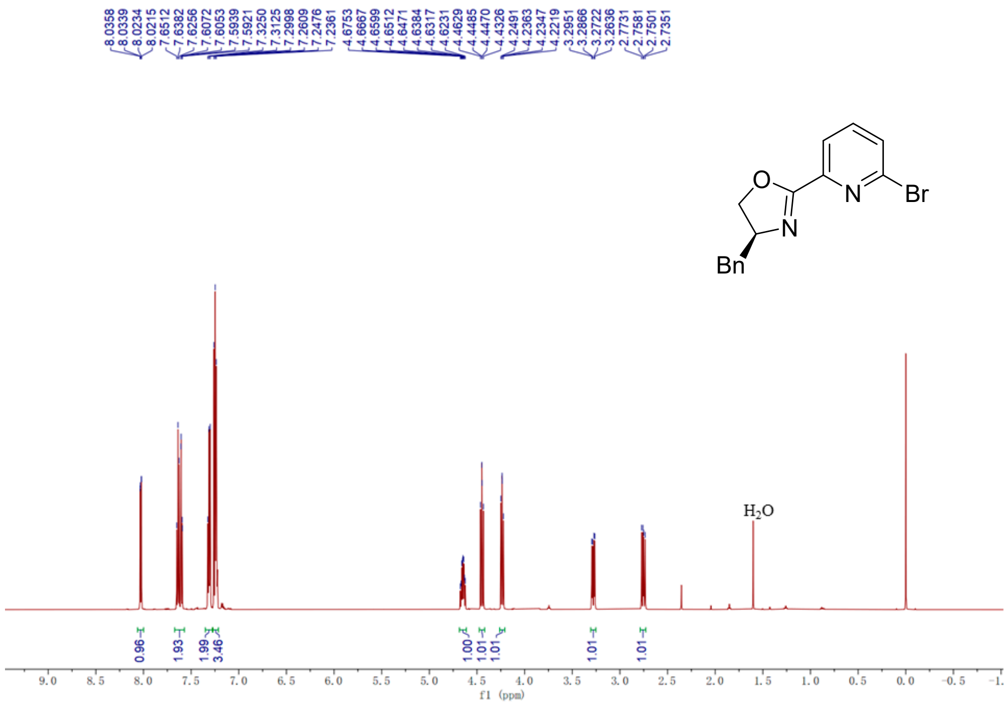

Figure **S25** $^{1}H$ NMR (600 MHz, $CDCl_3$) of **2g**

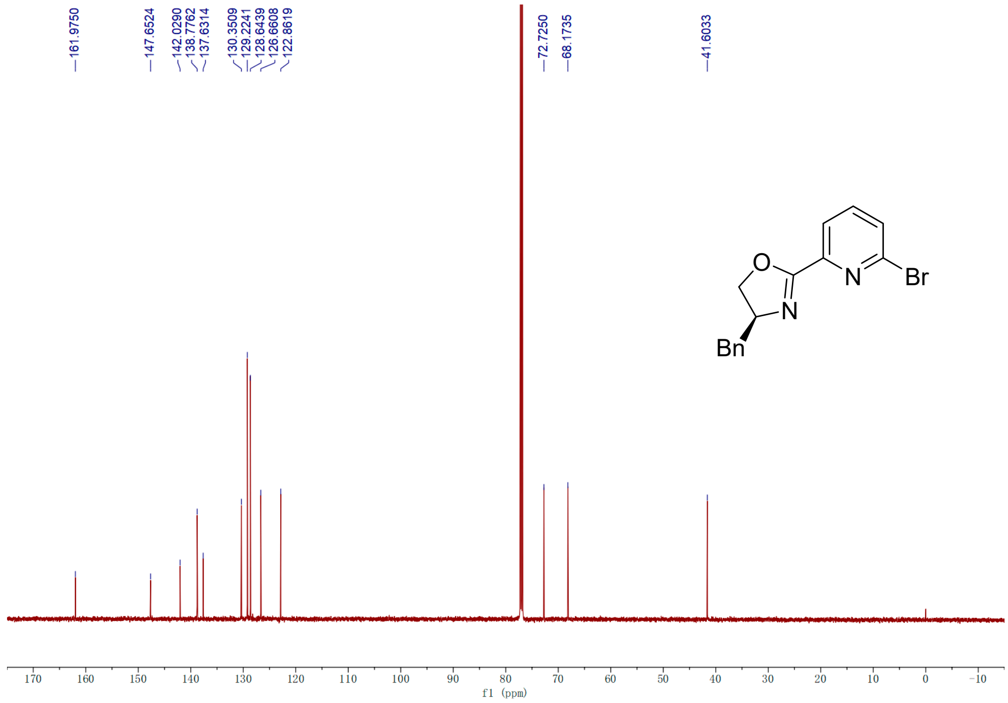

Figure **S26** $^{13}C\{^{1}H\}$ NMR (151 MHz, $CDCl_3$) of **2g**

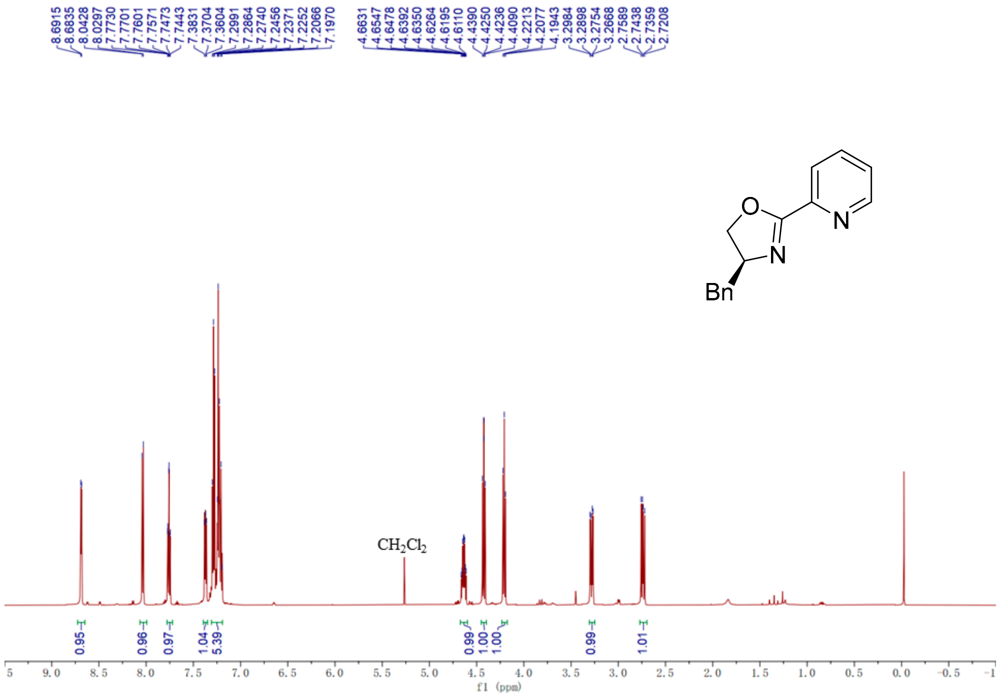

Figure **S27** $^{1}$H NMR (600 MHz, $CDCl_3$) of **2h**

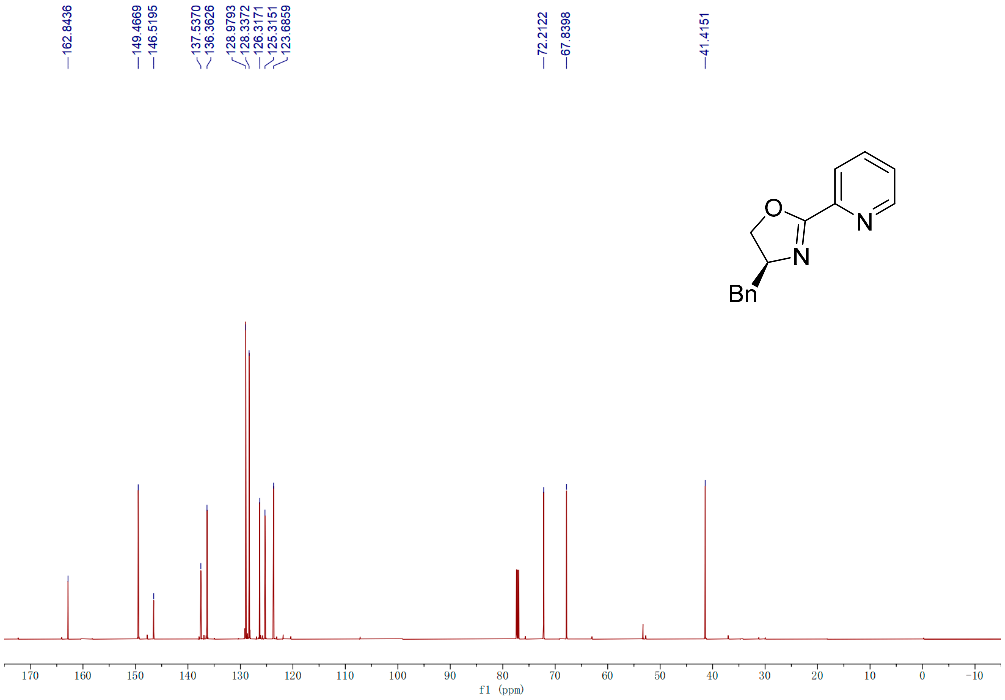

Figure **S28** $^{13}C\{^{1}H\}$ NMR (151 MHz, $CDCl_3$) of **2h**

## 2. Determination of Polymer Compositions

2.1 Calculation of monomer conversion:

$$\text{The conversion} = \frac{\text{The mass of the resulting polymer}}{\text{The total mass of the monomers used}} \times 100\%$$

2.2 Determination of polymer microstructure:

The microstructure of polyisoprene can be determined by NMR spectroscopy and the content of each structural unit can be calculated. Different microstructures correspond to different chemical shifts in the $^1$H and $^{13}$C NMR spectra.

**TableS1** The chemical shift value corresponding to the characteristic peak of polyisoprene

| 1,4-units | 3,4-units | 1,2-units |
|---|---|---|
| 5.14-5.10 ppm | 4.72-4.66 ppm | 5.80-5.60 ppm |

$$[\%1,4\text{-units}] = \frac{\text{I (5.14-5.10 ppm)}}{\text{I (5.14-5.10 ppm)} + \frac{\text{I (4.72-4.66 ppm)}}{2}} \quad \text{(Equation S1)}$$

$$[\%3,4\text{-units}] = \frac{\frac{\text{I (4.72-4.66 ppm)}}{2}}{\text{I (5.14-5.10 ppm)} + \frac{\text{I (4.72-4.67 ppm)}}{2}} \quad \text{(Equation S2)}$$

The *trans/cis*-1,4-units ratio can be determined by $^{13}$C NMR of the $-CH_3$ signals of *cis*-1,4 at 23.6 ppm and *trans*-1,4 at 16.2 ppm:

$$[\%\textit{trans}\text{-1,4-units}] = \frac{\text{I (16.2 ppm)}}{\text{I (16.2 ppm)} + \text{I (23.6 ppm)}} \quad \text{(Equation S3)}$$

$$[\%\textit{cis}\text{-1,4-units}] = \frac{\text{I (23.6 ppm)}}{\text{I (16.2 ppm)} + \text{I (23.6 ppm)}} \quad \text{(Equation S4)}$$

### 3. NMR Spectra and GPC Curve of Representative Polyisoprene

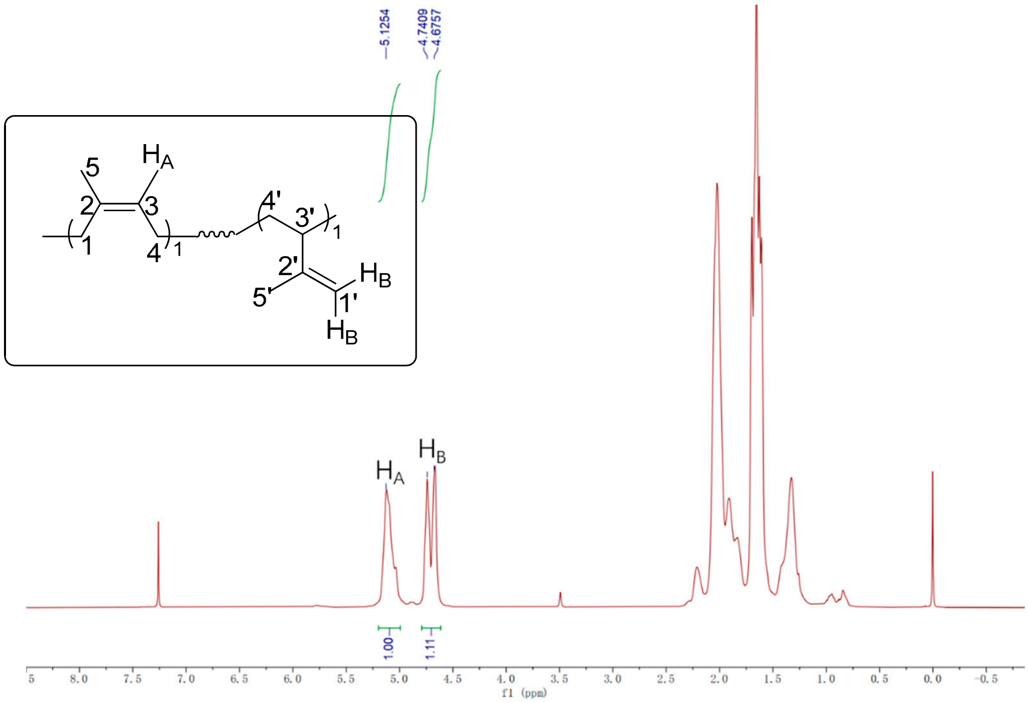


**Figure S29** $^{1}H$ NMR (600 MHz, $CDCl_3$) spectrum of Table 1, Entry 7

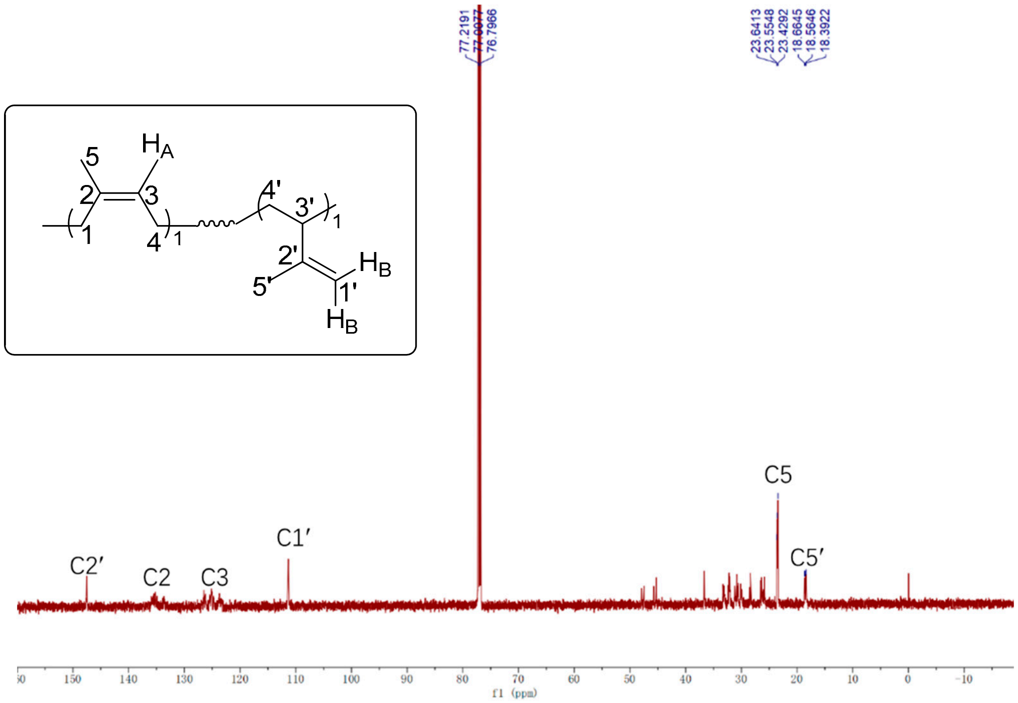


**Figure S30** $^{13}C$ NMR (151 MHz, $CDCl_3$) spectrum of Table 1, Entry 7

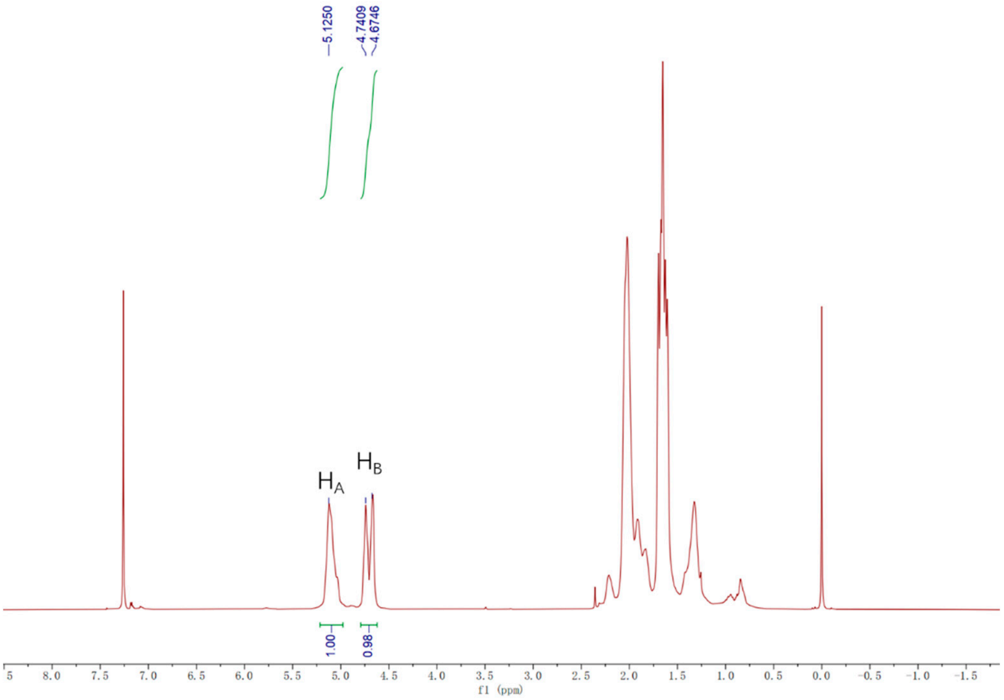


**Figure S31** $^{1}H$ NMR (600 MHz, $CDCl_3$) spectrum of Table 1, Entry 8

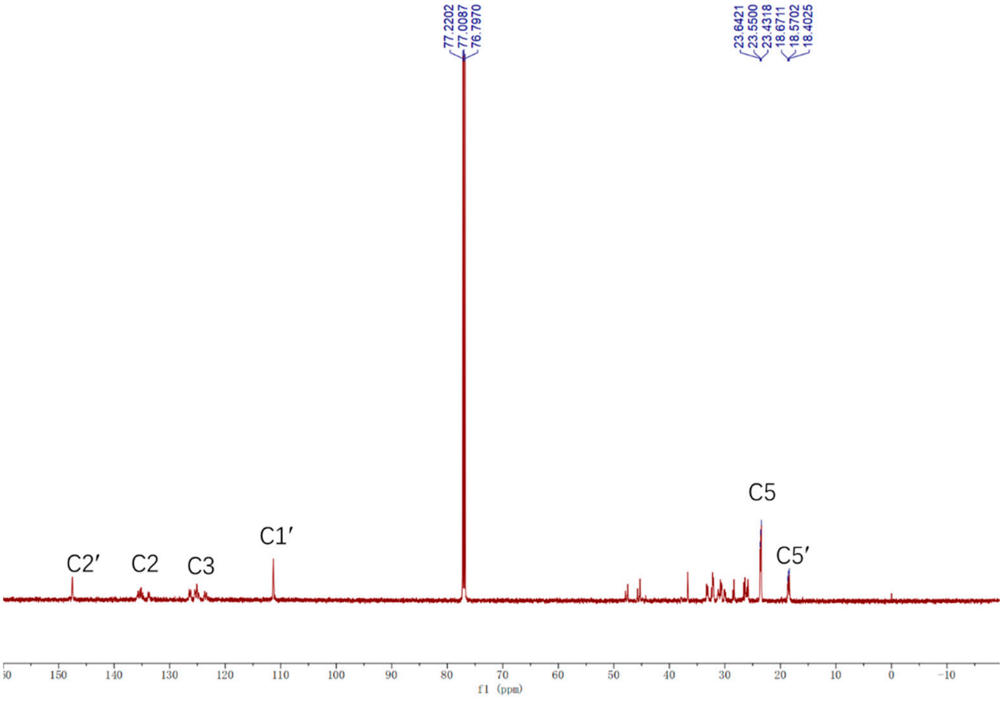


**Figure S32** $^{13}C$ NMR (151 MHz, $CDCl_3$) spectrum of Table 1, Entry 8

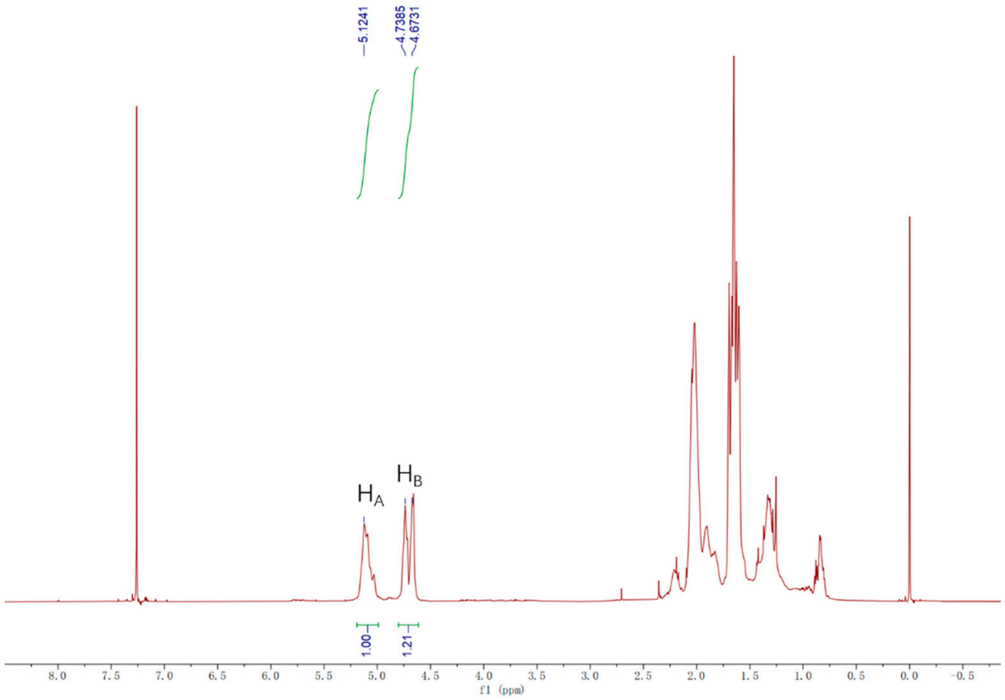


**Figure S33** $^{1}H$ NMR (600 MHz, $CDCl_3$) spectrum of Table 1, Entry 9

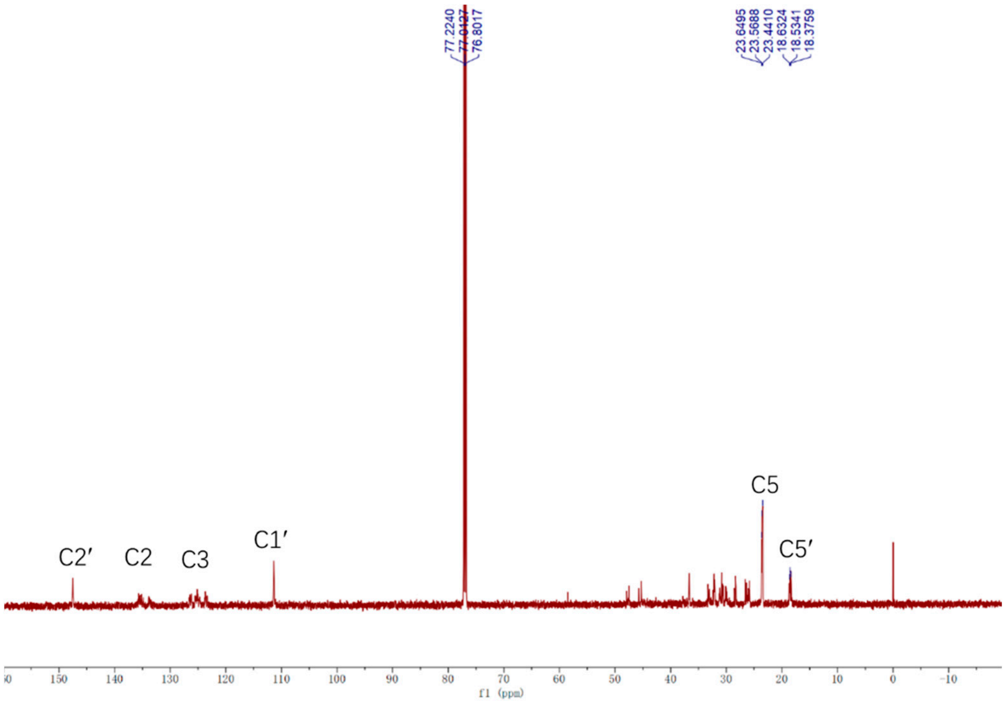


**Figure S34** $^{13}C$ NMR (151 MHz, $CDCl_3$) spectrum of Table 1, Entry 9

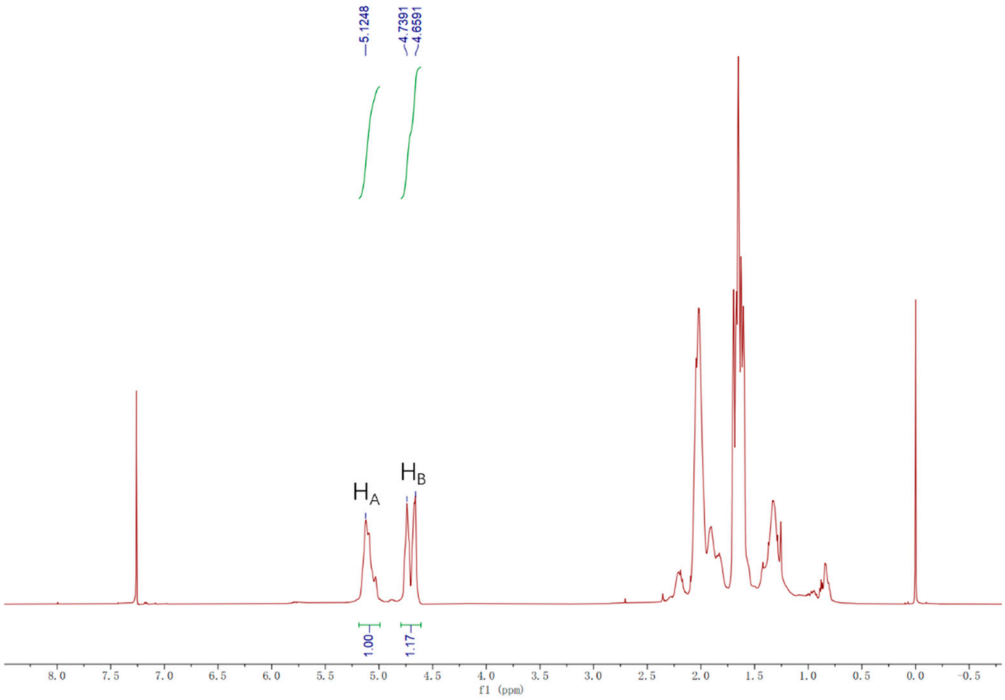


**Figure S35** $^{1}H$ NMR (600 MHz, $CDCl_3$) spectrum of Table 2, Entry 2

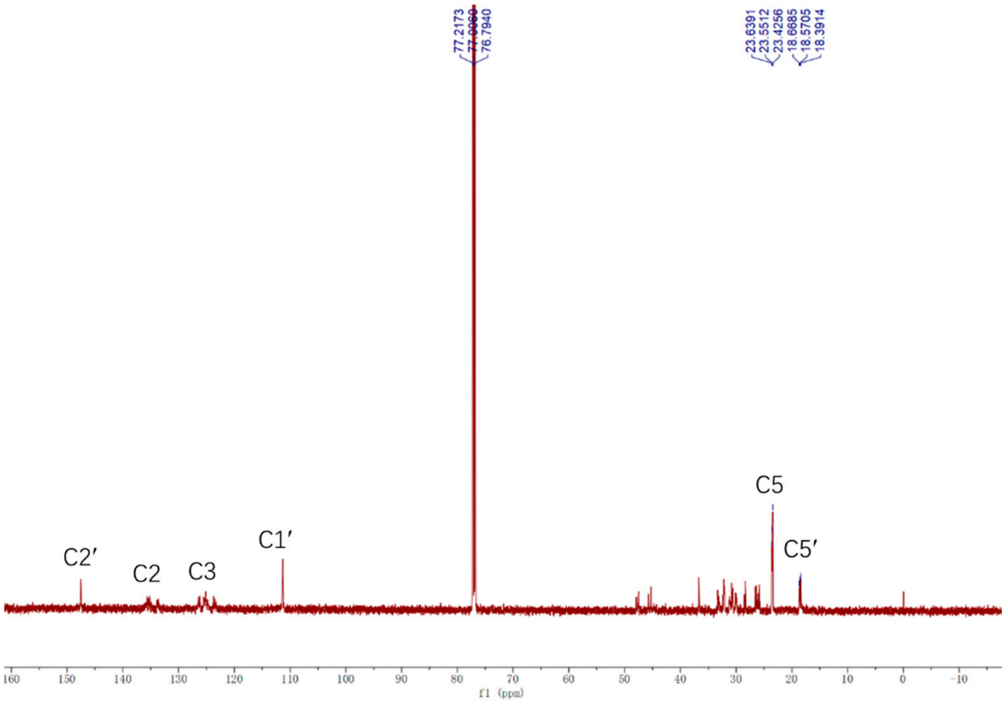


**Figure S36** $^{13}C$ NMR (151 MHz, $CDCl_3$) spectrum of Table 2, Entry 2

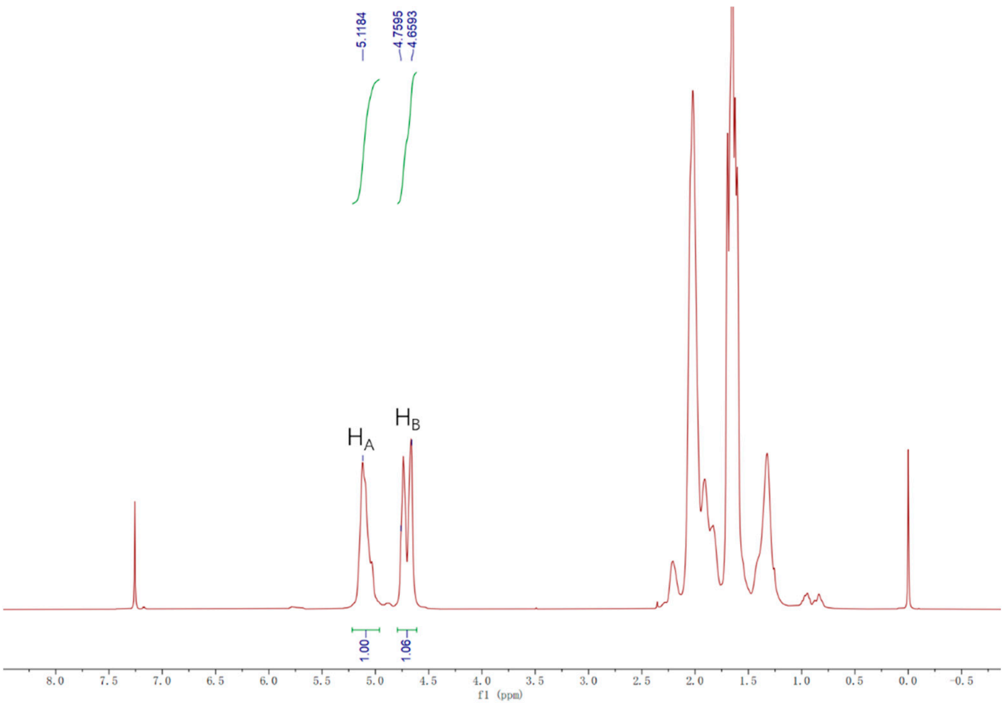


**Figure S37** $^{1}$H NMR (600 MHz, $CDCl_3$) spectrum of Table 2, Entry 7

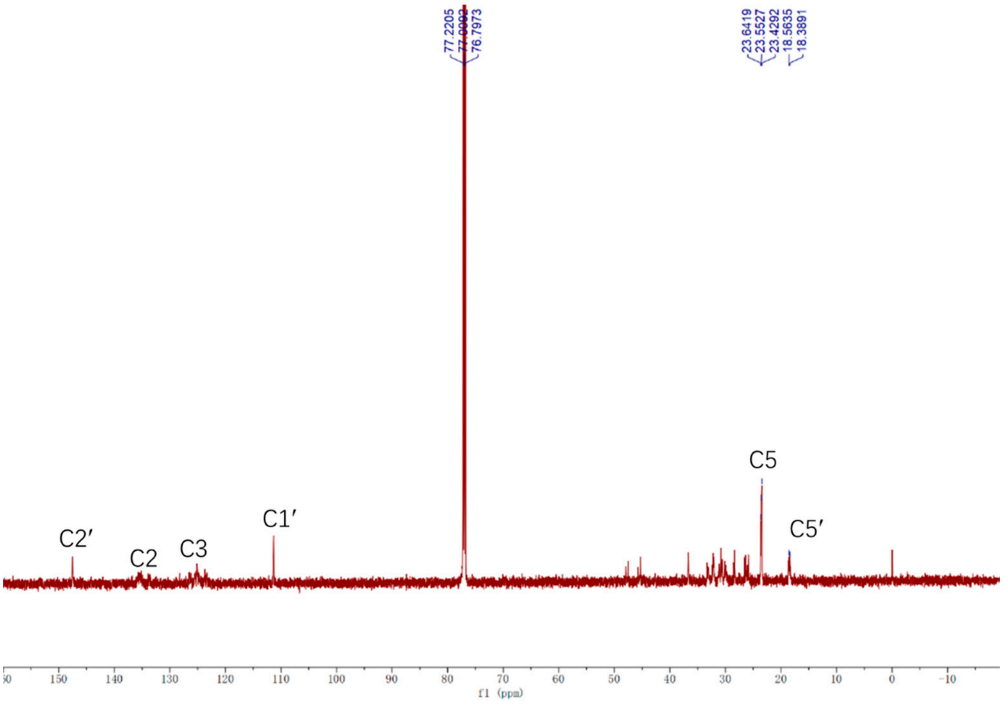


**Figure S38** $^{13}$C NMR (151 MHz, $CDCl_3$) spectrum of Table 2, Entry 7

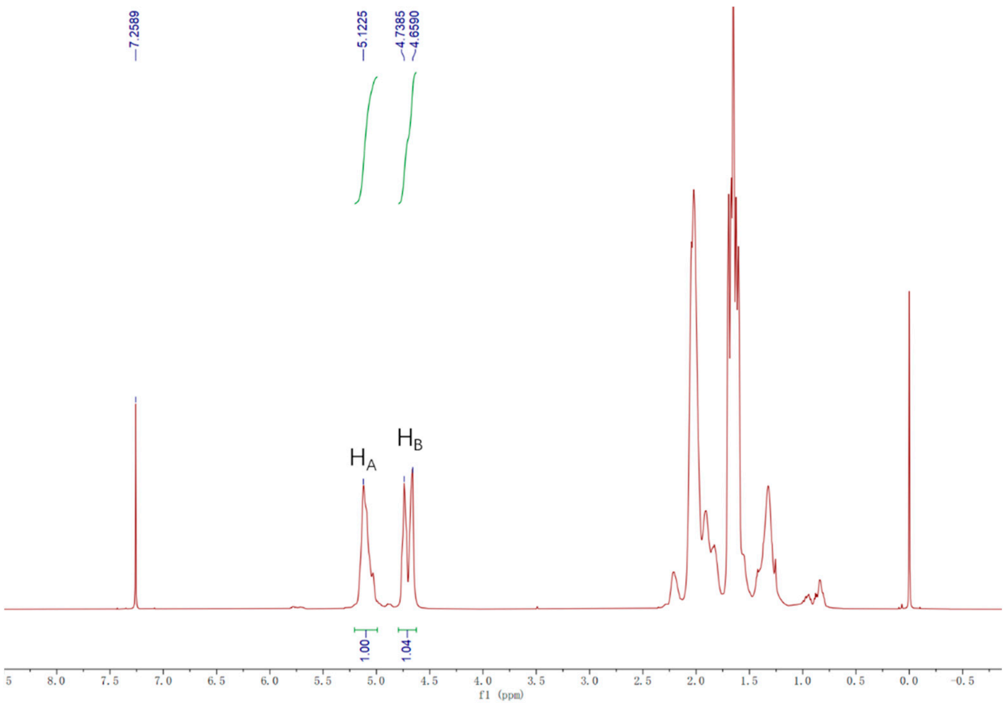


**Figure S39** $^{1}H$ NMR (600 MHz, $CDCl_3$) spectrum of Table 2, Entry 8

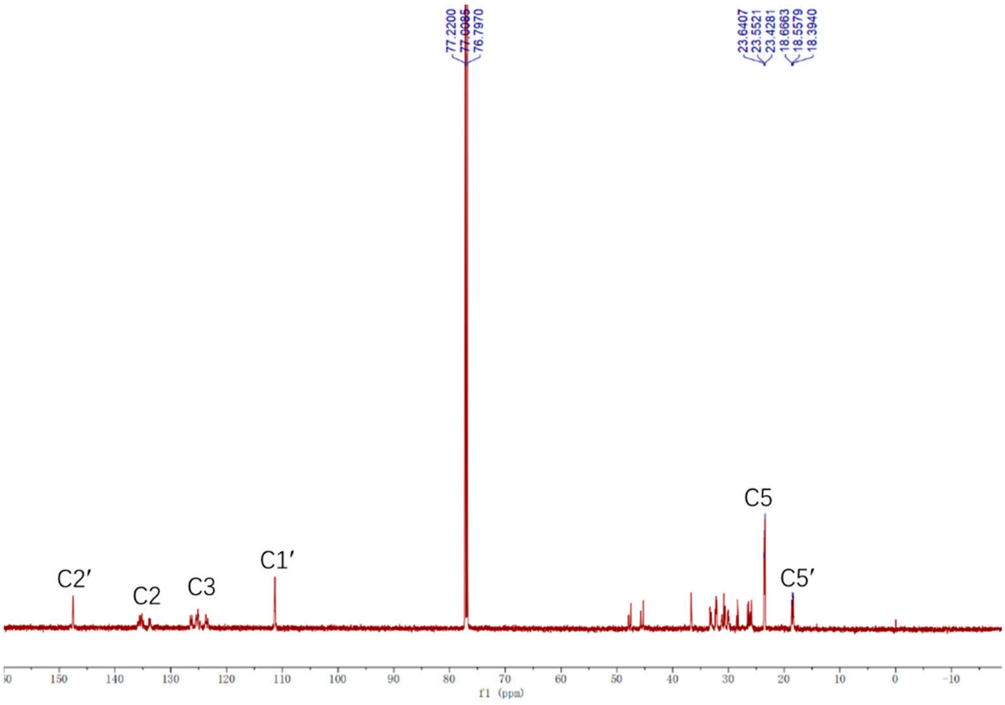


**Figure S40** $^{13}C$ NMR (151 MHz, $CDCl_3$) spectrum of Table 2, Entry 8

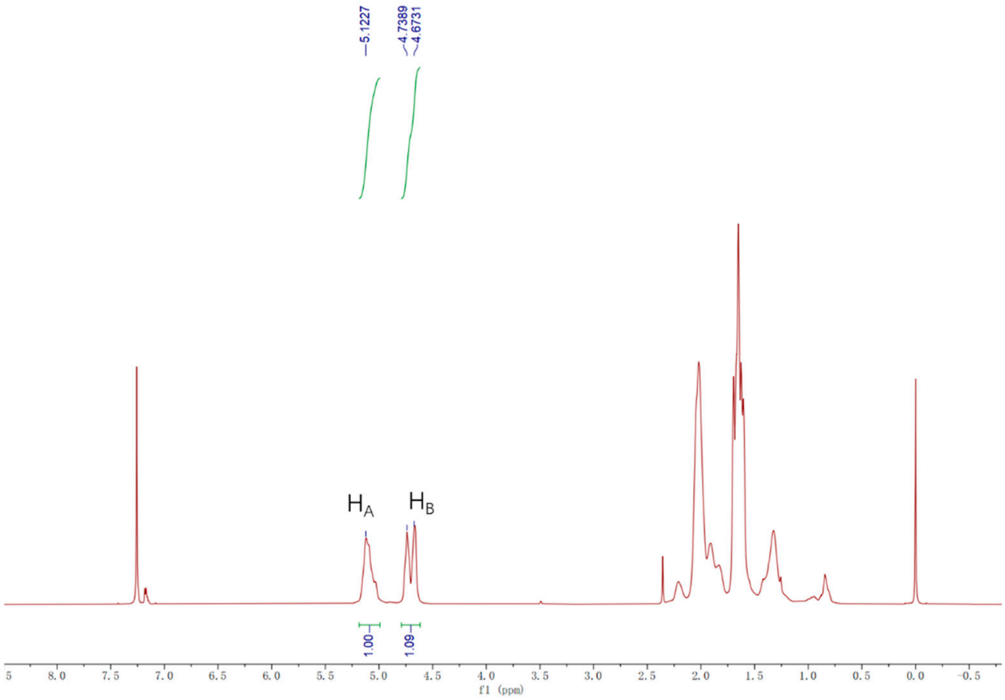


**Figure S41** $^{1}$H NMR (600 MHz, $CDCl_3$) spectrum of Table 3, Entry 4

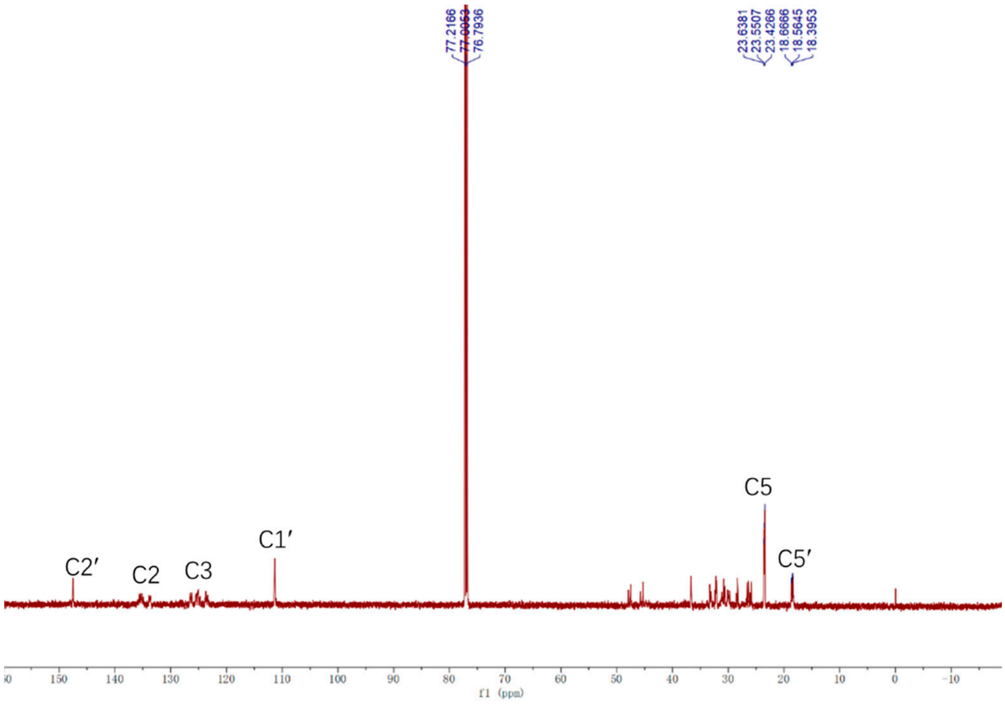


**Figure S42** $^{13}$C NMR (151 MHz, $CDCl_3$) spectrum of Table 3, Entry 4

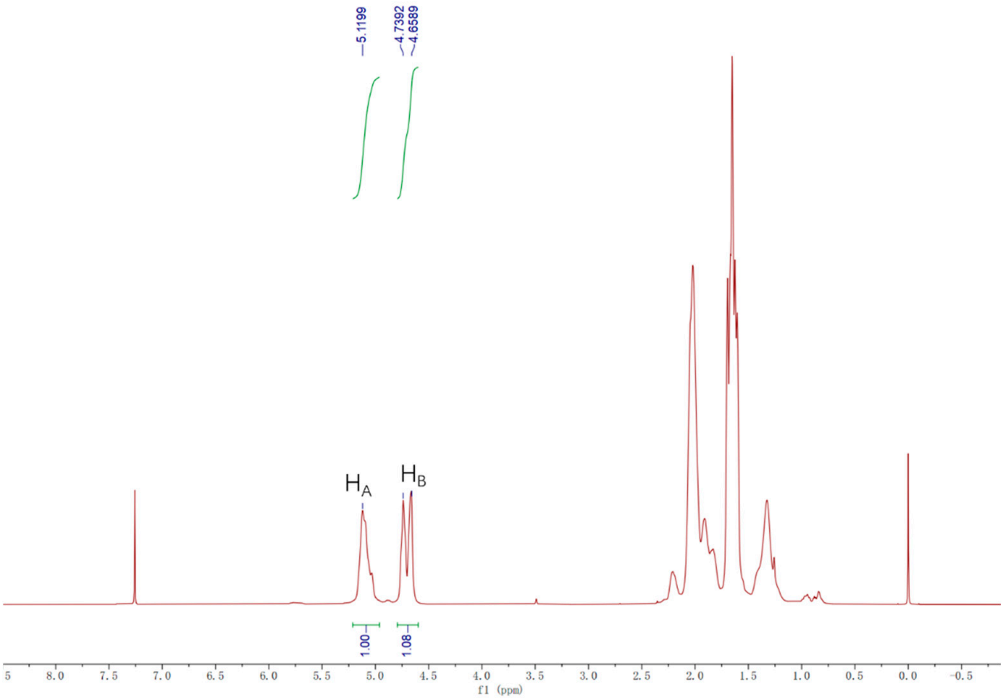


**Figure S43** $^{1}$H NMR (600 MHz, $CDCl_3$) spectrum of Table 3, Entry 7

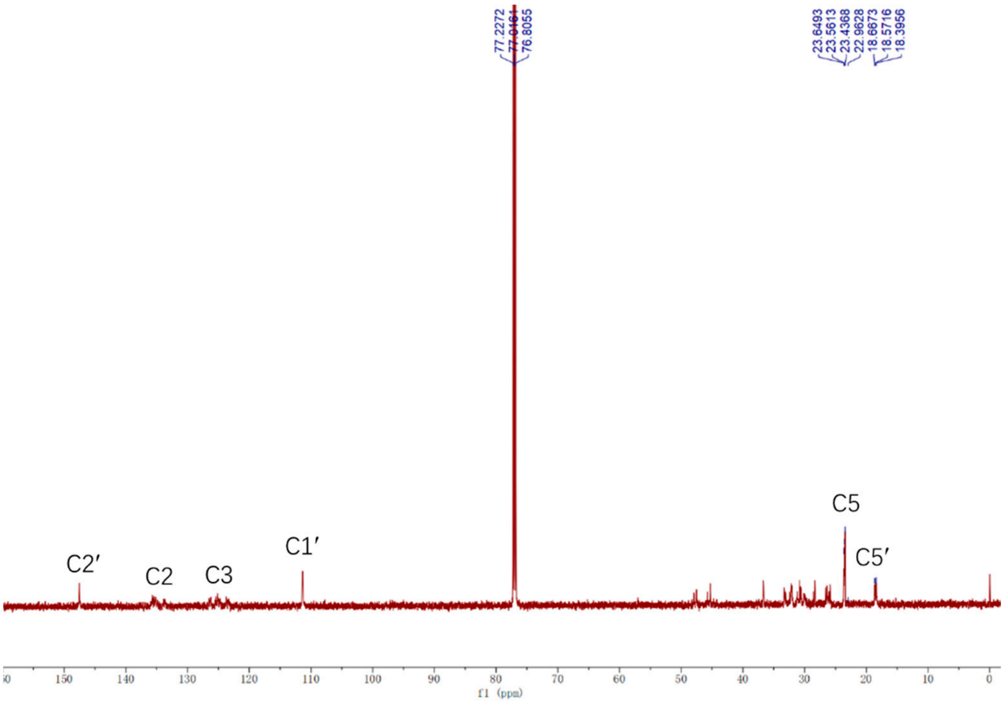


**Figure S44** $^{13}$C NMR (151 MHz, $CDCl_3$) spectrum of Table 3, Entry 7

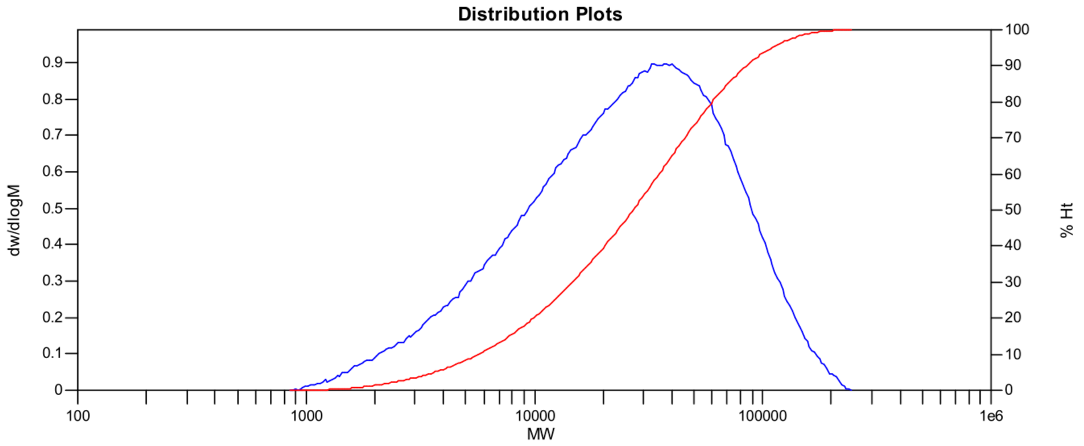


**MW Averages**

| Peak No | Mp | Mn | Mw | Mz | Mz+1 | Mv | PD |
|---|---|---|---|---|---|---|---|
| 1 | 37100 | 13261 | 37203 | 68864 | 98022 | 32956 | 2.80544 |

**Figure S45** GPC curve of the polyisoprene (Table 1, Entry 5)

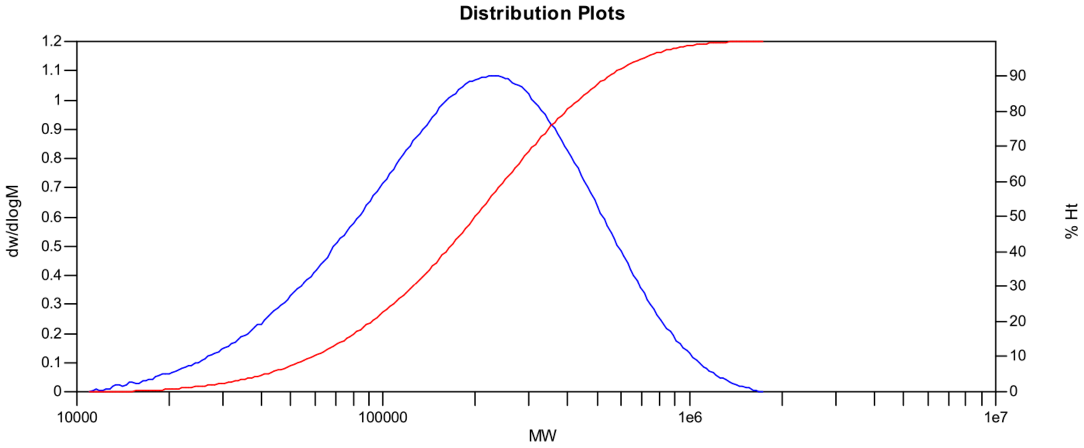


**MW Averages**

| Peak No | Mp | Mn | Mw | Mz | Mz+1 | Mv | PD |
|---|---|---|---|---|---|---|---|
| 1 | 232474 | 125458 | 257472 | 433957 | 622027 | 234354 | 2.05226 |

**Figure S46** GPC curve of the polyisoprene (Table 1, Entry 7; Table 2, Entry 1, and Table 3, Entry 1)

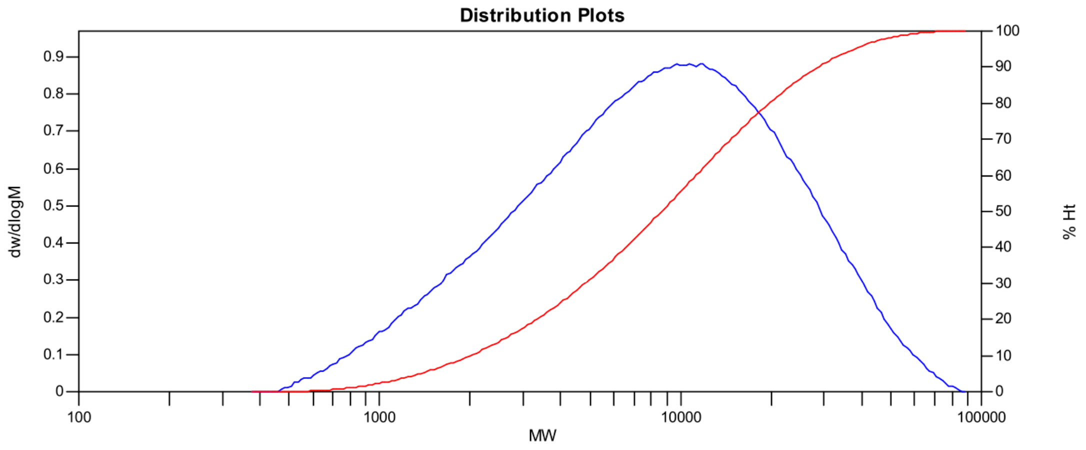


**MW Averages**

| Peak No | Mp | Mn | Mw | Mz | Mz+1 | Mv | PD |
|---|---|---|---|---|---|---|---|
| 1 | 10736 | 4733 | 12372 | 23668 | 34616 | 10928 | 2.61399 |

**Figure S47** GPC curve of the polyisoprene (Table 2, Entry 5)

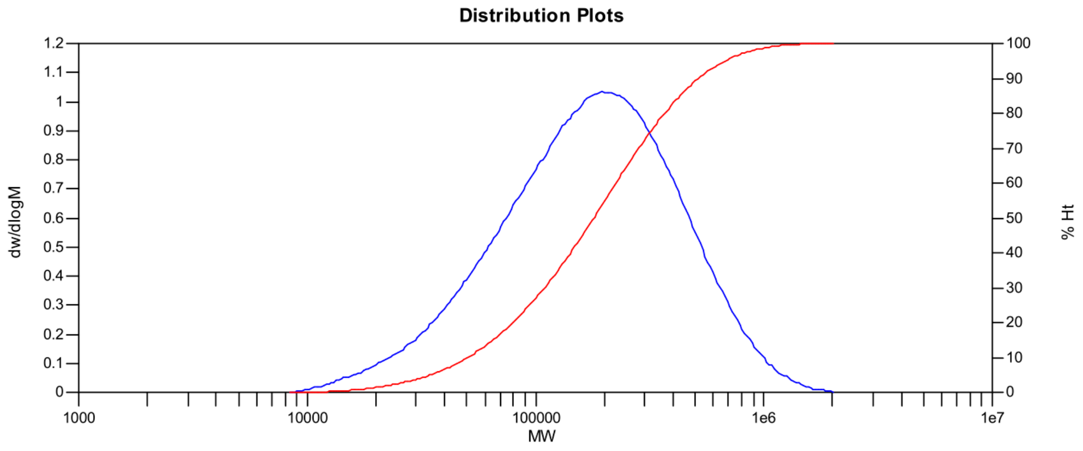


**MW Averages**

| Peak No | Mp | Mn | Mw | Mz | Mz+1 | Mv | PD |
|---|---|---|---|---|---|---|---|
| 1 | 195426 | 108825 | 240741 | 433291 | 657449 | 216779 | 2.21218 |

**Figure S48** GPC curve of the polyisoprene (Table 2, Entry 6)

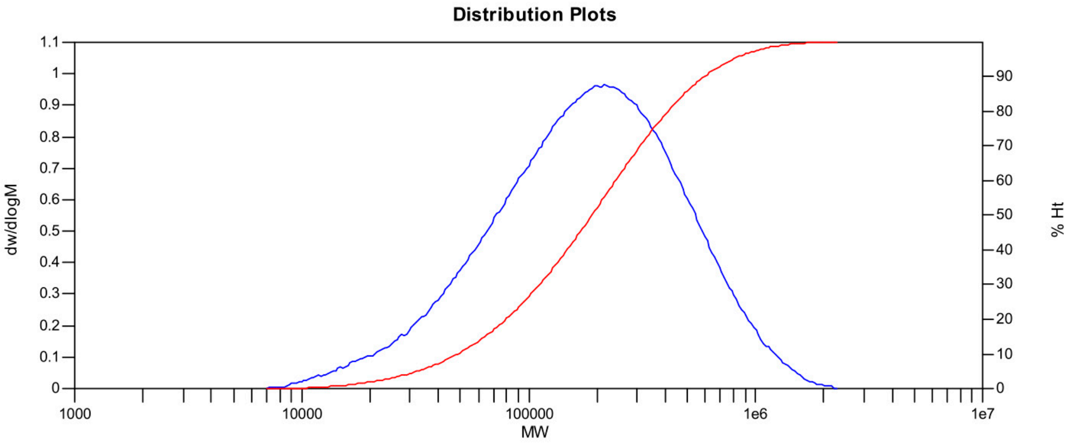


**MW Averages**

| Peak No | Mp | Mn | Mw | Mz | Mz+1 | Mv | PD |
|---|---|---|---|---|---|---|---|
| 1 | 215806 | 106774 | 265871 | 508251 | 782412 | 236270 | 2.49004 |

**Figure S49** GPC curve of the polyisoprene (Table 2, Entry 7)

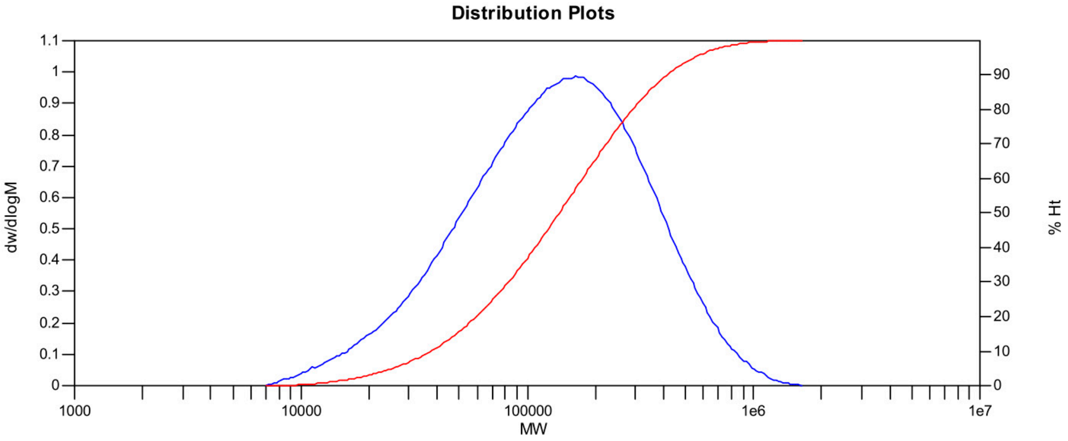


**MW Averages**

| Peak No | Mp | Mn | Mw | Mz | Mz+1 | Mv | PD |
|---|---|---|---|---|---|---|---|
| 1 | 164283 | 82083 | 189134 | 348215 | 528796 | 169386 | 2.30418 |

**Figure S50** GPC curve of the polyisoprene (Table 2, Entry 8)

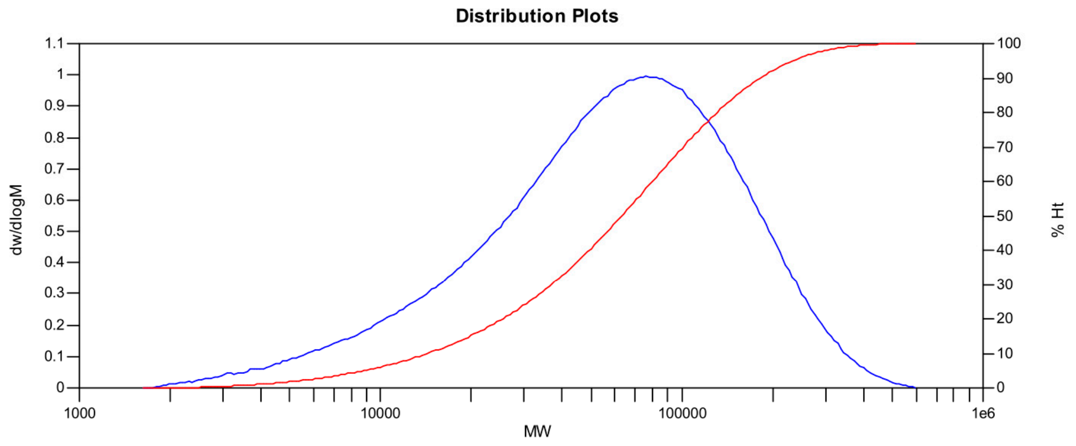


**MW Averages**

| Peak No | Mp | Mn | Mw | Mz | Mz+1 | Mv | PD |
|---|---|---|---|---|---|---|---|
| 1 | 76158 | 31474 | 83202 | 147711 | 212415 | 74555 | 2.64352 |

**Figure S51** GPC curve of the polyisoprene (Table 3, Entry 2)

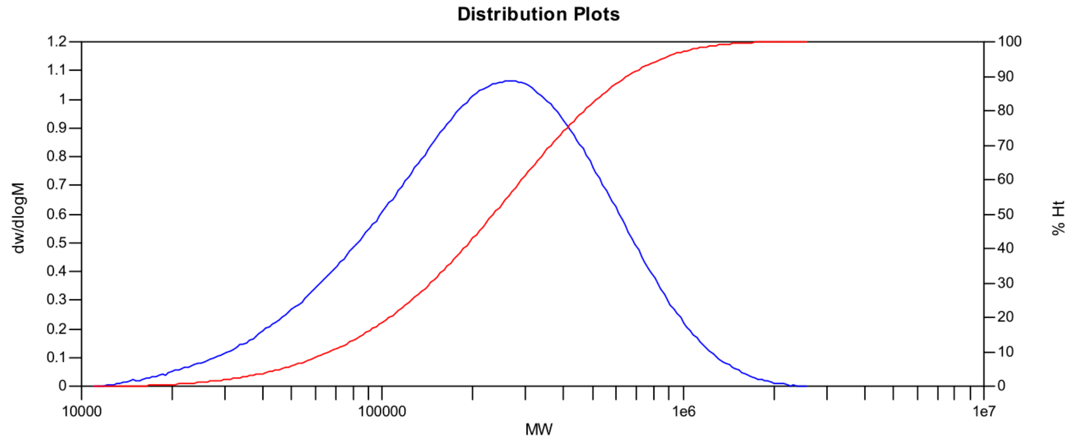


**MW Averages**

| Peak No | Mp | Mn | Mw | Mz | Mz+1 | Mv | PD |
|---|---|---|---|---|---|---|---|
| 1 | 269771 | 143690 | 306801 | 533523 | 788719 | 277860 | 2.13516 |

**Figure S52** GPC curve of the polyisoprene (Table 3, Entry 3)

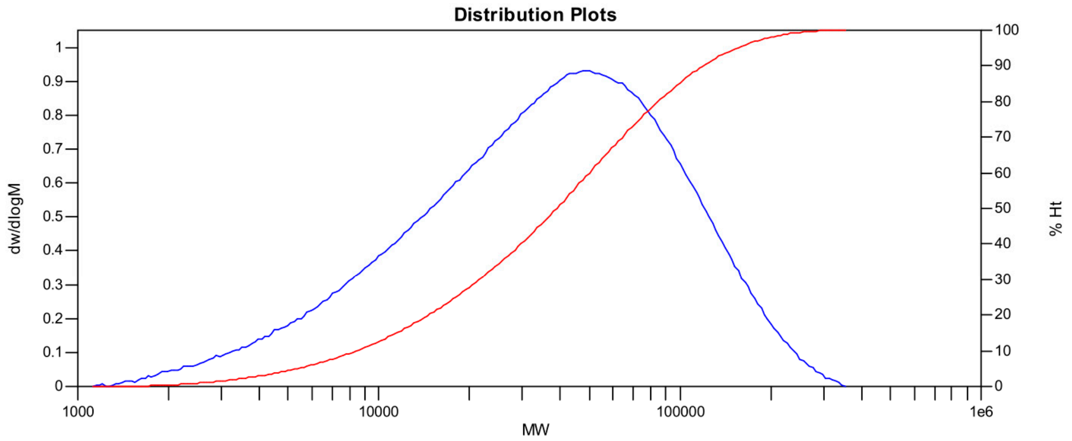


**MW Averages**

| Peak No | Mp | Mn | Mw | Mz | Mz+1 | Mv | PD |
|---|---|---|---|---|---|---|---|
| 1 | 49960 | 19239 | 52939 | 97439 | 139966 | 47022 | 2.75165 |

**Figure S53** GPC curve of the polyisoprene (Table 3, Entry 4)

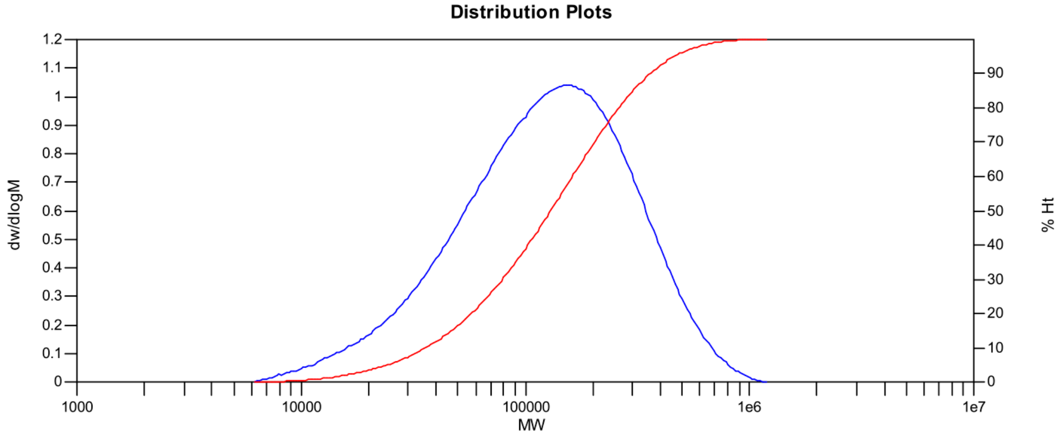


**MW Averages**

| Peak No | Mp | Mn | Mw | Mz | Mz+1 | Mv | PD |
|---|---|---|---|---|---|---|---|
| 1 | 156333 | 77436 | 168643 | 288565 | 413646 | 152829 | 2.17784 |

**Figure S54** GPC curve of the polyisoprene (Table 3, Entry 5)

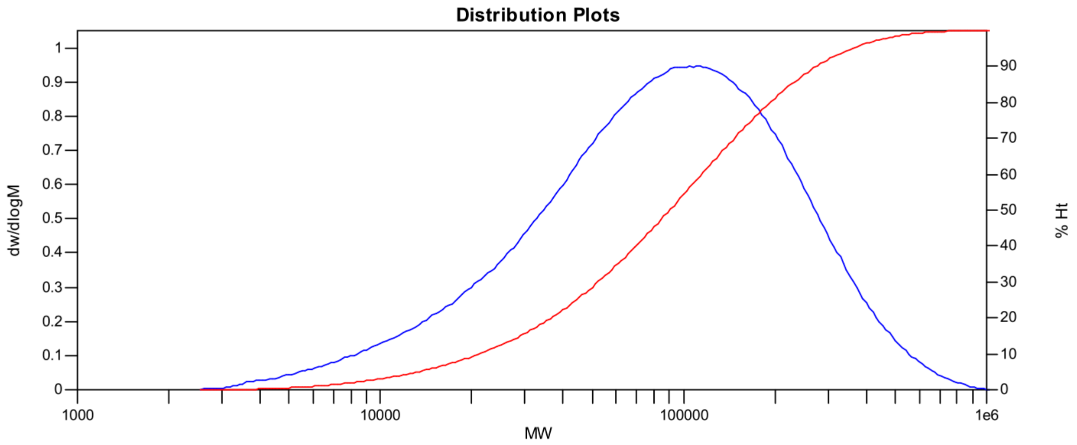


**MW Averages**

| Peak No | Mp | Mn | Mw | Mz | Mz+1 | Mv | PD |
|---|---|---|---|---|---|---|---|
| 1 | 110476 | 46583 | 123596 | 230777 | 345615 | 109956 | 2.65324 |

**Figure S55** GPC curve of the polyisoprene (Table 3, Entry 6)

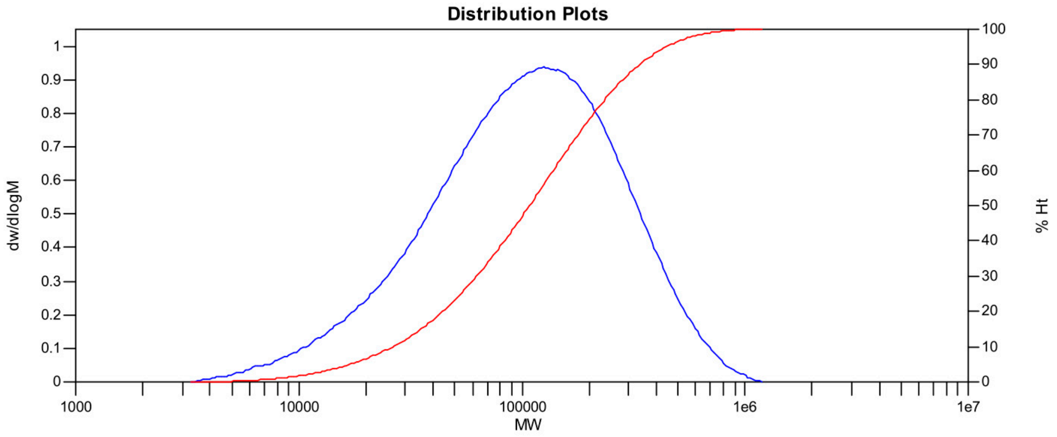


**MW Averages**

| Peak No | Mp | Mn | Mw | Mz | Mz+1 | Mv | PD |
|---|---|---|---|---|---|---|---|
| 1 | 125060 | 57834 | 150320 | 283050 | 424452 | 133587 | 2.59916 |

**Figure S56** GPC curve of the polyisoprene (Table 3, Entry 7)

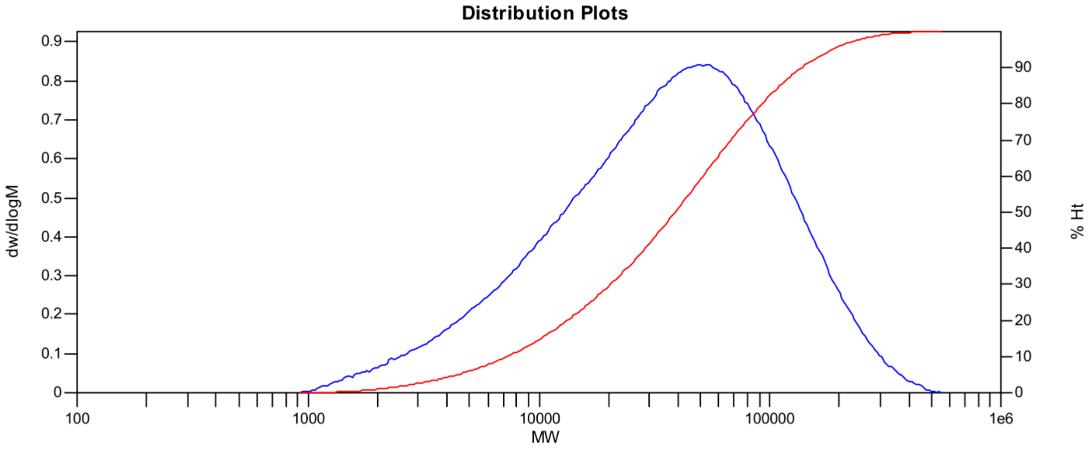


**MW Averages**

| Peak No | Mp | Mn | Mw | Mz | Mz+1 | Mv | PD |
|---|---|---|---|---|---|---|---|
| 1 | 53819 | 17004 | 58195 | 122664 | 190860 | 50351 | 3.42243 |

**Figure S57** GPC curve of the polyisoprene (Table 3, Entry 8)

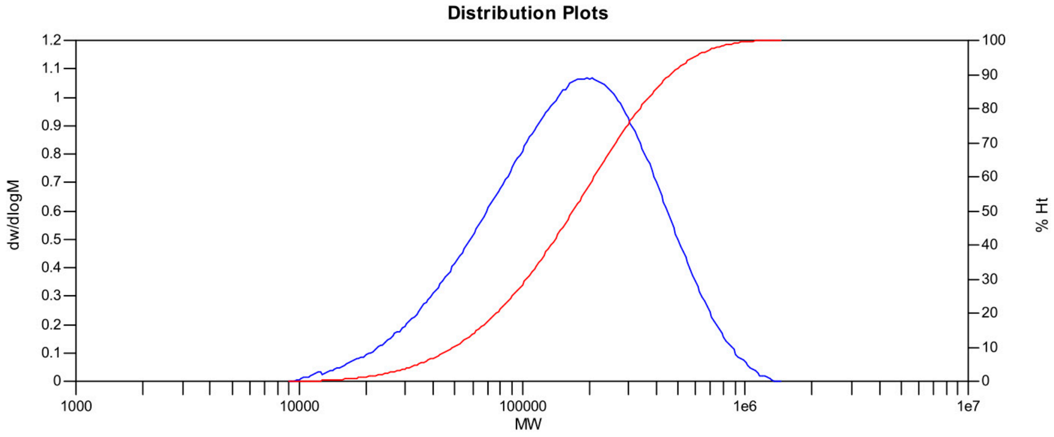


**MW Averages**

| Peak No | Mp | Mn | Mw | Mz | Mz+1 | Mv | PD |
|---|---|---|---|---|---|---|---|
| 1 | 195426 | 105454 | 218057 | 365348 | 516759 | 198480 | 2.06779 |

**Figure S58** GPC curve of the polyisoprene (Table 3, Entry 9)

## 4. FT-IR Spectra of Ligands and Complexes

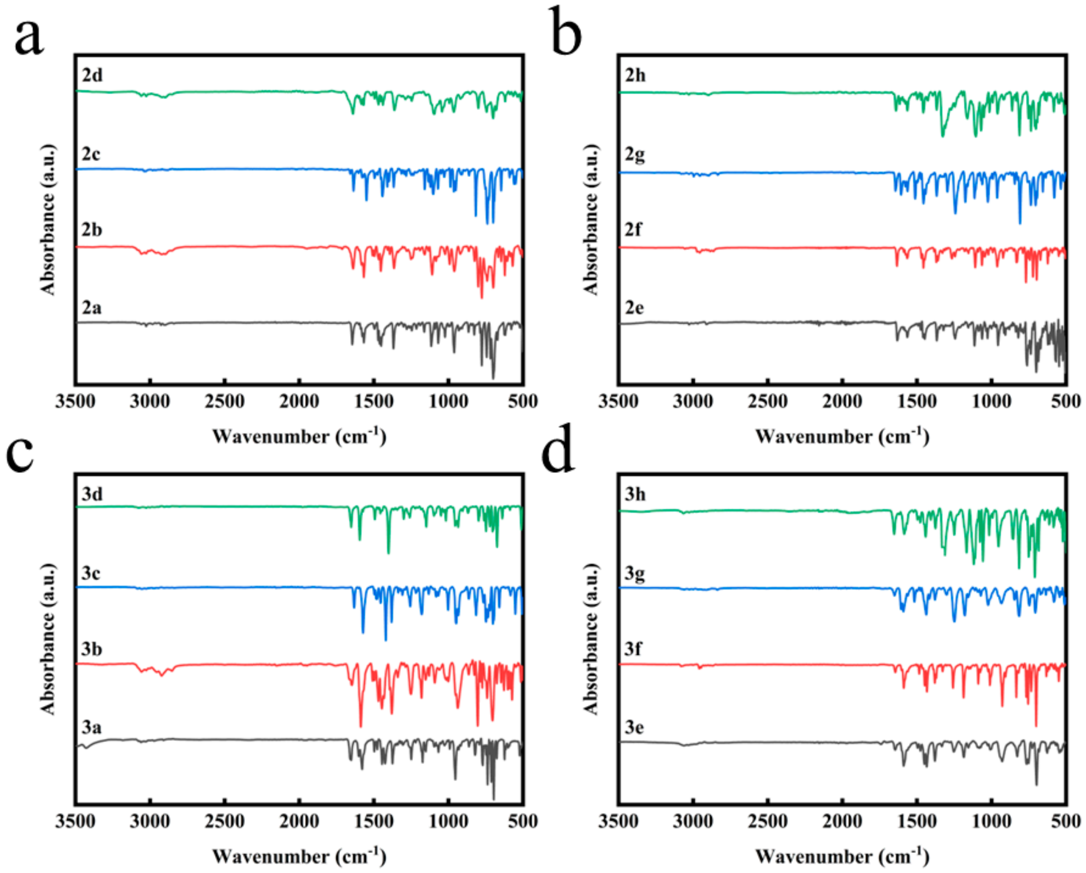


**Figure S59** The FT-IR spectra of ligands **2a-h** and complexes **3a-h**

## 5. X-ray Crystallographic Data

**Table S1.** Crystal data and structure refinement for complexes **3a** and **3d.**

| Identification code | **3a** | **3d** |
|---|---|---|
| Empirical formula | $C_{42}H_{36}Cl_2CoN_4O_2$ | $C_{44}H_{40}Cl_2CoN_4O_4$ |
| Formula weight | 758.58 | 818.63 |
| Temperature/K | 293(2) | 296 |
| Crystal system | orthorhombic | orthorhombic |
| Space group | $P2_12_12_1$ | $C222_1$ |
| a/Å | 7.51595(16) | 7.5048(3) |
| b/Å | 9.6603(2) | 19.9059(9) |
| c/Å | 50.8807(13) | 26.7070(14) |
| α/° | 90 | 90 |
| β/° | 90 | 90 |
| γ/° | 90 | 90 |
| Volume/Å$^3$ | 3694.25(15) | 3989.8(3) |
| Z | 4 | 4 |
| $\rho_{calc}$g/cm$^3$ | 1.364 | 1.363 |
| μ/mm$^{-1}$ | 5.303 | 0.612 |
| F(000) | 1572.0 | 1700.0 |
| Crystal size/mm$^3$ | 0.22 × 0.08 × 0.05 | 0.13 × 0.09 × 0.05 |
| Radiation | CuK$_\alpha$ (λ = 1.54184) | MoK$_\alpha$ (λ = 0.71073) |
| 2Θ range for data collection/° | 6.95 to 141.67 | 5.104 to 52.744 |
| Index ranges | $-8 \le h \le 8$, $-11 \le k \le 11$, $-62 \le l \le 59$ | $-8 \le h \le 9$, $-24 \le k \le 24$, $-33 \le l \le 33$ |
| Reflections collected | 15051 | 30699 |
| Independent reflections | 6483 [$R_{int}$ = 0.0456, $R_{sigma}$ = 0.0605] | 4092 [$R_{int}$ = 0.0944, $R_{sigma}$ = 0.0454] |
| Data/restraints/parameters | 6483/0/460 | 4092/0/250 |
| Goodness-of-fit on F$^2$ | 1.011 | 1.003 |
| Final R indexes [I>=2σ (I)] | $R_1$ = 0.0443, w$R_2$ = 0.0902 | $R_1$ = 0.0333, w$R_2$ = 0.0636 |
| Final R indexes [all data] | $R_1$ = 0.0572, w$R_2$ = 0.0970 | $R_1$ = 0.0531, w$R_2$ = 0.0693 |
| Largest diff peak/hole / e Å$^{-3}$ | 0.19/-0.26 | 0.16/-0.19 |
| Flack parameter | -0.025(4) | 0.016(10) |
